# Hyperacmosin R, a New Decarbonyl Prenylphloroglucinol with Unusual Spiroketal Subunit from *Hypericum acmosepalum*

**DOI:** 10.3390/molecules27185932

**Published:** 2022-09-13

**Authors:** Yonghui Ma, Xiaoyu Liu, Bo Liu, Pingping Li, Xinyue Suo, Tingting Zhu, Tengfei Ji, Jin Li, Xiaoxiu Li

**Affiliations:** 1 The Key Laboratory of Plant Stress Biology in Arid Land, College of Life Sciences, Xinjiang Normal University, Urumqi 830054, China; 2 State Key Laboratory of Bioactive Substance and Function of Natural Medicines, Institute of Materia Medica, Chinese Academy of Medical Sciences and Peking Union Medical College, Beijing 100050, China; 3 School of Pharmacy, Shenyang Medical College, Shenyang 110034, China; 4 Key Laboratory of Tibetan Medicine Research, Northwest Institute of Plateau Biology, Chinese Academy of Sciences, Xining 810008, China

**Keywords:** *Hypericum acmosepalum*, PPAPs, 5,8-spiroketal, hepatoprotective activity

## Abstract

Two previously undescribed polycyclic polyprenylated acylphloroglucinols, hyperacmosins R-S (**1**–**2**), were obtained from the aerial parts of *Hypericum acmosepalum*. Their structures were elucidated by extensive spectroscopic analysis and electronic circular dichroism calculation (ECD). Compound **1** featured an unprecedented 5,8-spiroketal subunit as well as the loss of C-2′ carbonyl in the phloroglucinol ring. In addition, compounds **1** and **4** showed weak hepatoprotective activity against paracetamol-induced HepG2 cell damage at 10 μm. The plausible biosynthetic pathway of **1** was proposed via a retro-Clasisen reaction and decarboxylation.

## 1. Introduction

Polycyclic polyprenylated acylphloroglucinols (PPAPs), the prominent secondary metabolites of the genus *Hypericum*, are a group of structurally fascinating and synthetically challenging natural products, which possess highly oxygenated acylphloroglucinol-derived cores decorated with isoprenyl, geranyl, or other substituted side chains [1,2]. To date, more than 900 natural PPAPs with diverse carbon skeletons have been isolated. Apart from their structures, these compounds exhibit a broad range of biological activities, such as acetylcholinesterase inhibitory activity [3], cytotoxic activity [3,4,5], anti-inflammatory activity [6], phosphodiesterase-4 inhibitory activity [7], CYP3A4 enzyme inhibitory activity [8], antiplasmodial activity [9], and anti-HIV activity [10].

**Figure 1:**
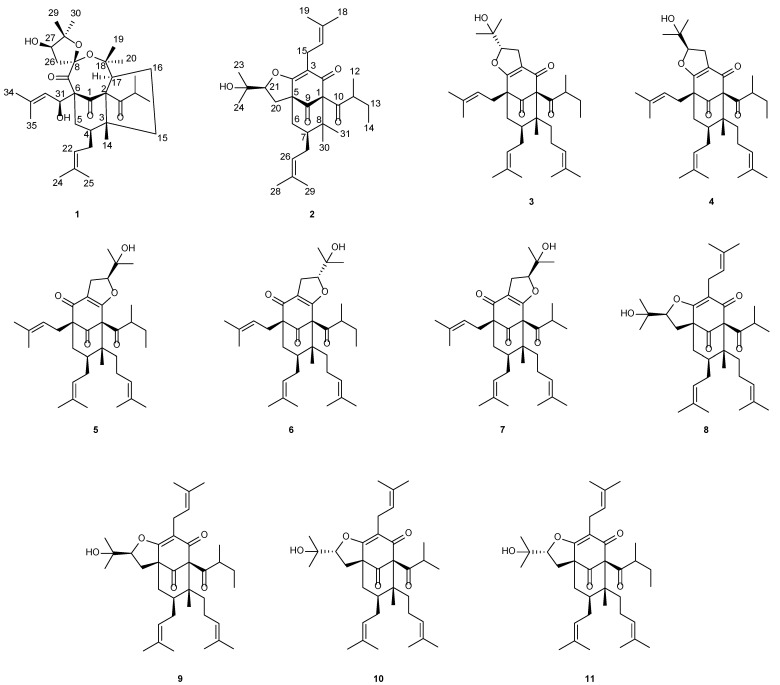
Chemical structures of compounds **1**–**11**.

*Hypericum acmosepalum* is distributed in Guangxi, Yunnan, Sichuan, and Guizhou provinces in China. As a kind of traditional Chinese medicine, it has been used to treat hepatitis and relieve swelling and inflammation [11]. Our previous chemical investigations into this plant resulted in the isolation of many bioactive PPAPs with diverse carbon scaffolds, and some of them showed hepatoprotective and neuroprotective activities [12,13,14,15,16]. In order to obtain more of this type of molecules, our continuing study of this plant led to the identification of two new PPAPs, hyperacmosins R-S (**1**–**2**), as well as nine known ones (Figure 1). It is worth mentioning that compound **1** possesses a rare 5,8-spiroketal subunit, together with the loss of C-2′ carbonyl in the phloroglucinol ring. Herein, the details of the isolation, structural elucidation, and the plausible biosynthetic ways of compound **1** are reported.

## 2. Results and Discussion

Hyperacmosin R (**1**) was obtained as colorless oil, [α]^20^_D_ -230.5 (c 0.1375, MeOH). HR-ESI-MS spectrum (*m/z* 573.3786 [M+H]^+^, calcd. 573.3786) revealed its molecular formula to be C_34_H_52_O_7_, indicating nine degrees of unsaturation, and its IR data implied the existence of hydroxyl (3533 cm^−1^) and carbonyl (1716 cm^−1^). The ^1^H NMR spectrum of **1** (Table 1) showed characteristic resonances for one isopropyl [*δ*_H_ 2.90 (1H, m), 1.07 (3H, d, *J* = 6.4 Hz), 1.41 (3H, d, *J* = 6.4 Hz) ], one hydroxyl [*δ*_H_ 2.66 (1H, brs)], two olefinic protons [*δ*_H_ 4.87 (1H, brs) and 5.31 (1H, brs)], and nine singlet methyls (*δ*_H_ 0.88, 089, 1.15, 1.22, 1.33, 1.60, 1.62, 1.68, 1.78). Combined with its HSQC and HMBC spectra, the ^13^C NMR data of **1** (Table 1) revealed 34 signals, including three nonconjugated carbonyls (*δ*_C_ 202.5, 206.2, 218.7) and four olefinic carbons (*δ*_C_ 120.4, 124.7, 133.4, 136.0), accounting for five indices of hydrogen deficiency. Apart from the aforementioned 7 carbons, the remaining 27 carbon signals were assigned to two oxygenated tertiary carbons (*δ*_C_ 85.4 and 90.1), three oxygenated secondary carbons (*δ*_C_ 113.1, 76.1, and 36.9), three quaternary carbons (*δ*_C_ 78.8, 58.2, and 67.1), five methylenes (*δ*_C_ 36.3, 35.4, 27.5, 30.9, and 44.5), three methines (*δ*_C_ 39.3, 43.4, and 54.7), and 11 methyls (*δ*_C_ 18.1, 18.4, 18.6, 20.5, 22.9, 23.8 × 2, 26.1, 26.2, 26.5, and 28.0). Combined with the remaining four indices of hydrogen deficiency, the above data indicated that **1** might be a tetracyclic PPAP analogue.

**Figure 2:**
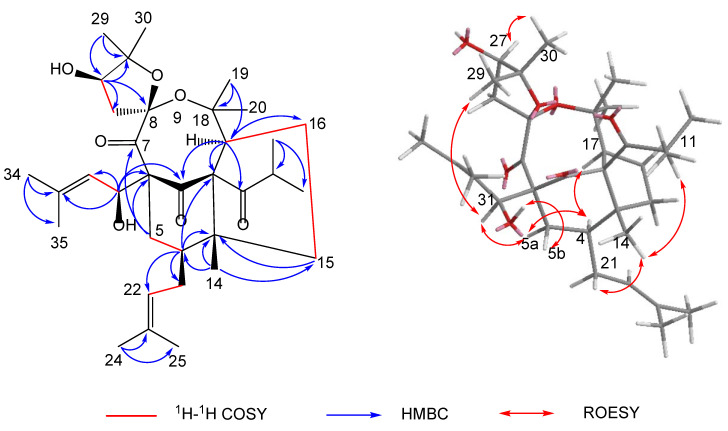
Key 2D NMR correlations of hyperacmosin R (**1**).

The polycyclic core structure of **1** was established by a comprehensive analysis of the 2D NMR spectral data. The HMBC correlations (Figure 2) from H-4 (*δ*_H_ 1.98) to C-2/C-3/C-5/C-6, from H_2_-5 (*δ*_H_ 1.25/2.45) to C-1/C-3/C-4/C-6, from H-17 (*δ*_H_ 3.04) to C-1/C-2/C-3/C-15/C-16, and from H_2_-15 (*δ*_H_ 1.34/1.89) to C-2/C-3/C-4/C-16/C-17, along with ^1^H-^1^H COSY correlations of H-4/H_2_-5, H_2_-15/H_2_-16, and H_2_-16/H-17, established the cyclohexanone (B-ring) and cyclopentane moieties (A-ring), respectively. Moreover, the HMBC correlations (Figure 2) from H-27 (*δ*_H_ 4.00) to C-8/C-26/C-28/C-29/C-30, from H_3_-29 (*δ*_H_ 1.22)/H_3_-30 (*δ*_H_ 1.33) to C-27/C-28, and from H_2_-26 (*δ*_H_ 2.37) to C-7/C-8/C-27/C-28, combined with the presence of two oxygen-bearing carbons C-8 (*δ*_C_ 113.1) and C-28 (*δ*_C_ 90.1), implied the presence of the 2,2-dimethyl-3-hydroxy-furan unit (D-ring). Considering one remaining unsaturation as well as the diagnostic ketal carbon C-8 (*δ*_C_ 113.1), the fourth circle (C-ring) was formed via an oxygen connecting C-8 and C-18. Moreover, an isobutyryl group and two isoprenyl groups were attached to C-2, C-4, and C-6, respectively, supported by the HMBC cross-peaks from H-11 (*δ*_H_ 2.90) to C-2, from H_2_-21 (*δ*_H_ 1.70/2.08) to C-3/C-4/C-5, and from H-31 (*δ*_H_ 3.20) to C-1/C-5/C-6/C-7. It is noteworthy that the abnormal chemical shift of C-31 (*δ*_C_ 36.9) was much lower than the normal value. By viewing the 3D model of compound 1, it might be attributed to the shielding effect of the C-1 carbonyl group. 

The relative configuration of **1** was confirmed on the basis of the ROESY spectrum (Figure 2). The ROESY correlations of H-5b/HO-31, H-31/H-5a, H-5a/H-4, Me-14/H_2_-21, and Me-14/H-11 revealed that HO-31, Me-14, and the two isoprenyl groups were on the same side and were assigned as *β*-orientation. In addition, the ROESY cross-peaks of H-4/H-17 revealed that H-17 was *α*-oriented. Subsequently, the obvious correlation of Me-29/H-31 and H-27/Me-30 demonstrated that Me-29 and Me-30 were at the upper side of the C-ring. Thus, the structure of **1** was determined, as shown in Figure 2.

**Figure 3:**
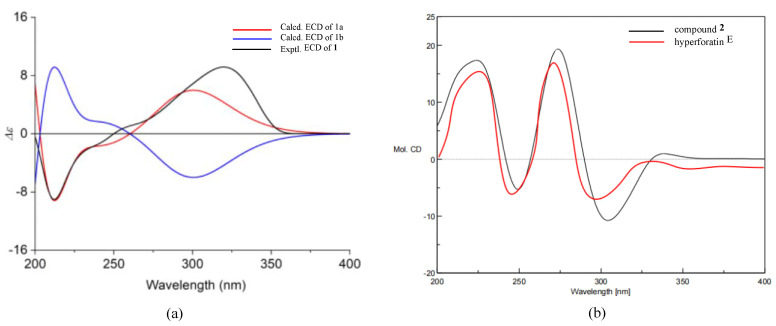
(**a**) Calculated and experimental ECD spectra of **1**. (**b**) The ECD spectra of **2** and hyperforatin E.

The absolute configuration of **1** was elucidated by electronic circular dichroism (ECD) calculation, using the time-dependent density functional theory (TD-DFT). A pair of enantiomers, (2*R*, 3*R*, 4*S*, 6*R*, 8*R*, 17*S*, 27*R*, 31*S*)-**1a** and (2*S*, 3*S*, 4*R*, 6*S*, 8*S*, 17*R*, 27*S*, 31*R*)-**1b**, were calculated for the ECD spectra based on the known relative configuration of **1**. The ECD spectrum (Figure 3) of **1** was in sufficient agreement with **1a**. Thus, the absolute configuration of **1** was assigned as 2*R*, 3*R*, 4*S*, 6*R*, 8*R*, 17*S*, 27*R*, and 31*S*.

**Figure 4:**
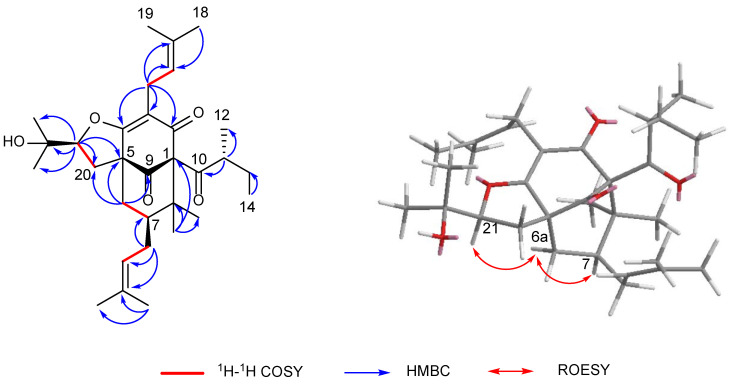
Key 2D NMR correlations of hyperacmosin S (**2**).

Hyperacmosin S (**2**) was obtained as a colorless oil. The molecular formula was established as C_31_H_46_O_5_ according to its HRESIMS data (*m/z* 499.3417 [M+H]^+^, calcd. 499.3418), indicating nine degrees of unsaturation. The ^1^H NMR spectrum (Table 1) showed characteristic signals assignable to two olefinic protons [*δ*_H_ 5.06 (1H, t, *J* = 7.4 Hz) and 4.94 (1H, t, *J* = 7.0 Hz)], a sec-butyl group [*δ*_H_ 1.71 (1H, m), 1.27 (1H, m), 1.63 (1H, m), 0.77 (3H, t, *J* = 7.4 Hz), 1.08 (3H, d, *J* = 6.4 Hz)], and eight singlet methyls (*δ*_H_ 1.71, 1.69, 1.65, 1.56, 1.39, 1.27, 1.22, 1.05). The ^13^C NMR spectrum (Table 1) of **2** displayed 31 carbon resonances, including three carbonyl carbons (*δ*_C_ 208.7, 204.7, 193.0) and four olefinic carbon (*δ*_C_ 133.6, 132.6, 122.4, 121.3). Detailed analysis of the 1D and 2D NMR (Figure 4) of **2** indicated compound **2** shared the same structure with garsubellin B, except for the values of optical rotation [**2**: [*α*]^24^_D_ +32.3 (*c* 0.52, EtOH); garsubellin B: [*α*]^24^_D_ -36 (*c* 0.6, EtOH)] [17]. This indicated that compound **2** is the enantiomer of garsubellin B. Finally, the absolute configuration of **2** was established by comparing its experimental ECD spectrum with that of hyperforatin E (Figure 3) [3].

Based on the comparison of their NMR and MS data with the literature values, nine known PPAPs were identified as furoadhyperforin isomer A (**3**) [18], furoadhyperforin isomer B (**4**) [18], furohyperforin isomer 2a (**5**) [19], furohyperforin isomer 2b (**6**) [19], furohyperforin isomer 2 (**7**) [8], furohyperforin (**8**) [8], furoadhyperforin (**9**) [8], hypercohin E (**10**) [20], and hypercohin F(**11**) [20], respectively.

All of the isolated compounds were evaluated for their hepatoprotective activities against paracetamol-induced HepG2 cell damage, and glutathione was used as the positive control. As shown in Table 2, hyperacmosin R (**1**) and furoadhyperforin isomer B (**4**) exhibited weak hepatoprotective activity at 10 μm. 

**Figure 5:**
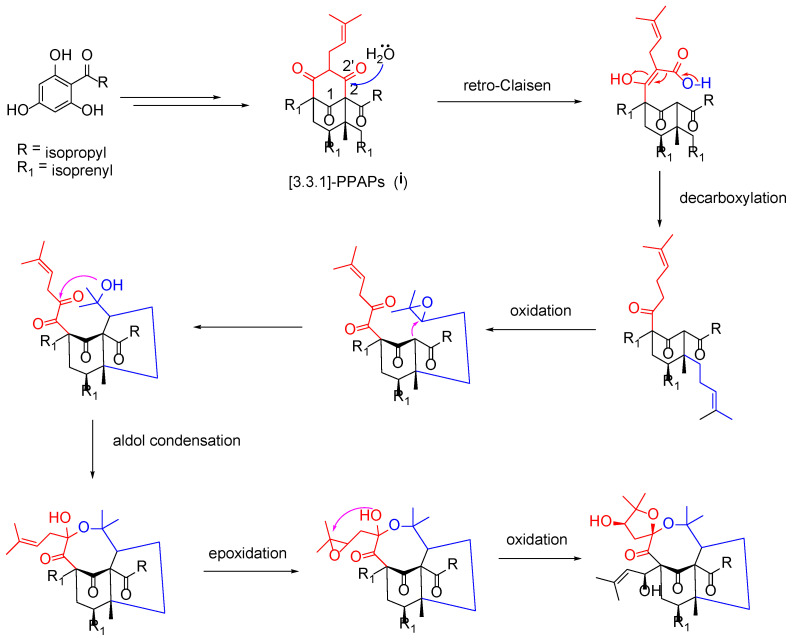
Plausible biosynthetic pathway for **1**.

Structurally, hyperacmosin R (**1**) possesses a rare 5,8-spiroketal subunit, together with the loss of C-2′ carbonyl in the phloroglucinol ring. The plausible biogenetic pathway of hyperacmosin R (**1**) was proposed in Figure 5. Starting from 2,4,6-trihydroxybenzophenone, the intermediate (**i**), which possesses bicyclo [3.3.1] nonane-2,4,9 trione core, is formed via a series of prenylation and cyclization reactions [1]. The carbonyl at C-2′ was likely degenerated through a retro-Clasisen reaction and decarboxylation [21]. Moreover, the 5,8-spiroketal subunit might be formed, successively, via oxidation, aldol condensation, epoxidation, and an intramolecular cyclization reaction.

## 3. Materials and Methods

### 3.1. General Experimental Procedures

Optical rotations were measured on a JASCO P-2000 polarimeter (JASCO Inc. Tokyo, Japan). UV spectra were measured on a JASCO V650 spectrophotometer (JASCO Inc.). The CD spectra were measured on a JASCO J-815 CD spectrometer (JASCO Inc.). IR spectra were recorded on a Nicolet 5700 FT-IR spectrometer (Thermo Nicolet, Waltham, MA, USA). NMR spectra were acquired with VNS-400 spectrometers and VNS-500 spectrometers (Varian Inc., Palo Alto, CA, USA). HRESI-MS spectra were collected on an Agilent 1100 series LC/MSD ion trap mass spectrometer (Agilent Technologies Ltd, Santa Clara, CA, USA). Preparative HPLC was performed on a Shimadzu LC-6AD (SHIMADZU Inc. Tokyo, Japan) instrument with an SPD-20A detector, using an YMC-Pack ODS-A column (2 × 25 cm, 5 µm). Column chromatography was performed with silica gel (200–300 mesh, Qingdao Marine Chemical Inc., Qingdao, China) and ODS (50 µm, YMC, Kyoto, Japan). Chiral AD-H column (4.6 mm × 250 mm, 5 µm, Daicel, Osaka, Japan); TLC was carried out on glass precoated silica gel GF254 plates. Spots were visualized under UV light or by spraying with 10% sulfuric acid in EtOH, followed by heating.

### 3.2. Plant Materials

The air-dried aerial parts of *H. acmosepalum* were collected from Lijiang, Yunnan Province (100°11′ E; 26°11′ N), People’s Republic of China, in July 2016. Lin Ma was responsible for the identification of the plant, based on the comparison with the specimen preserved in the Institute of Botany, Chinese Academy of Sciences. A voucher specimen (No. ID-S-2764) was deposited in the Institute of Materia Medica, Chinese Academy of Medical Sciences.

### 3.3. Extraction and Isolation

The air-dried aerial parts of *H. acmosepalum* (15.0 kg) were extracted by 95% ethanol (150 L × 3 times) under reflux. The crude extract was suspended in H_2_O and partitioned with petroleum ether. The petroleum ether extract (510.0 g) was separated on a silica gel column with petroleum ether/EtOAc (100:0 to 0:100, *v*/*v*) to gain five fractions (Fr.1–5). Fr.3 (95.2 g) was further purified by chromatography on a diol column, eluting with petroleum ether/EtOAc (100:0 to 0:100, *v*/*v*) to yield fourteen fractions (Fr.3.1–Fr.3.14). Fr.3.10 (11.5 g) was chromatographed over a C_8_ silica column eluted with a gradient system of MeOH−H_2_O (85% to 100%, *v*/*v*) to give 7 fractions (Fr.3.10.1–Fr.3.10.7), Fr.3.10.6 was sequentially purified by semi-preparative HPLC (MeOH-H_2_O, 90:10) to yield **3** (2.2 mg), **4** (25.0 mg), **6** (17.3 mg), and **7** (6.2 mg). Fr.3.11 (36.4 g) was fractionated using a silica column with CH2Cl2-EtOAc (100:0 to 0:100, *v*/*v*) as eluent to give 7 fractions (Fr.3.11.1–Fr.3.11.7). Fr.3.11.5 was sequentially purified by semi-preparative HPLC (MeOH-H_2_O, 90:10, *v*/*v*) to yield **2** (4.2 mg), **8** (10.1 mg), **9** (16.3 mg), **10** (12.5 mg), and **11** (5.1 mg). Fr.3.11.6 was chromatographed over a C8 silica column eluted with MeOH−H_2_O (95:5, *v*/*v*) to yield **1** (102.0 mg). Fr.3.11.7 was sequentially purified by semi-preparative HPLC (MeOH-MeCN-H_2_O, 70:15:15) to yield **5** (10.3 mg) (flow chart, see Figure S21).

### 3.4. Structural Elucidation

Hyperacmosin R (**1**)**:** colorless oil; [*α*]^20^_D_ -230.5 (*c* 0.14, MeOH); UV (MeOH) *λ*_max_ (log *ε*) 204 (4.05) nm; ECD (MeOH) *λ*_max_ (∆*ε*) 212 (−9.02), 320 (9.18) nm; IR *υ*_max_ 3533, 2977, 2929, 1716, 1687, 1450, 1380 cm^−1^; ^1^H and ^13^C NMR data, see Table 1; HRESIMS *m/z* 573.3786 [M + H]^+^ (calcd. for C_34_H_53_O_7_, 573.3786).

Hyperacmosin S (**2**)**:** colorless oil; [*α*]^20^_D_ +92.3 (*c* 0.05, MeOH); UV (MeOH) *λ*_max_ (log *ε*) 203 (4.45), 271 (4.43) nm; ECD (MeOH) *λ*_max_ (∆*ε*) 224 (17.32), 250 (−5.31), 274 (19.29), 304 (−10.79), 339 (0.93) nm; IR *υ*_max_ 3446, 2975, 2930, 1730, 1626, 1452, 1369 cm^−1^; ^1^H and ^13^C NMR data, see Table 1; HRESIMS *m/z* 499.3417 [M + H]^+^ (calcd. for C_31_H_47_O_5_, 499.3418).

### 3.5. Hepatoprotection Bioassays (In Vitro)

The hepatoprotective effects of compounds **1**–**11** were determined by a (MTT) colorimetric assay in HepG2 cells. Each cell suspension of 2 × 10^4^ cells in 200 µL of RPMI 1640 containing fetal calf serum (10%), penicillin (100 U/mL), and streptomycin (100 µg/mL) was placed in a 96-well microplate and precultured for 24 h at 37 ℃ under 5% CO_2_ atmosphere. Fresh medium (100 µL) containing bicyclol and test samples was added, respectively, and the cells were cultured for 1 h. The cultured cells were exposed to 16 mM paracetamol for 24 h. Then, 100 µL of 0.5 mg/mL MTT was added to each well, after the withdrawal of the culture medium, and incubated for additional 4 h. The resulting formazan was dissolved in 150 µL DMSO after aspiration of the culture medium. The optical density (OD) of the formazan solution was measured on a microplate reader at 570 nm. Percentage inhibition was calculated as: inhibition (%) = [OD (sample) − OD (control)]/[OD (normal) − OD (control)] × 100%. 

## 4. Conclusions

In summary, a detailed chemistry investigation of *H.* *acmosepalum* led to the identification of 11 PPAPs, including 2 previously undescribed ones, hyperacmosins R-S (**1**–**2**). Especially, hyperacmosin R (**1**) possesses a rare 5,8-spiroketal subunit, together with the loss of C-2′ carbonyl in the phloroglucinol ring. All the isolates were evaluated for their hepatoprotective activities. Among them, hyperacmosin R (**1**) and furoadhyperforin isomer B (**4**) exhibited weak capabilities against paracetamol-induced HepG2 cell damage at 10 μm. Furthermore, the plausible biosynthetic pathway of hyperacmosins R (**1**) was proposed. This study enriched the members and the structural diversity of PPAPs from *H.* *acmosepalum.*

## Figures and Tables

**Table 1:** ^1^H NMR and ^13^C NMR data for compounds **1**–**2**.

No	1 ^a^		2 ^b^	
	*δ*_H_ (*J* in Hz)	*δ*_C_, Type	*δ*_H_ (*J* in Hz)	*δ*_C_, Type
1		206.2, C		82.3, C
2		78.8, C		193.0, C
3		58.2, C		116.6, C
4	1.98, brs	39.3, CH		173.0, C
5	2.45, m; 1.25, m	36.3, CH_2_		59.7, C
6		67.1, C	2.03, m; 1.49, m	38.6, CH_2_
7		202.5, C	1.49, m	42.5, CH
8		113.1, C		46.6, C
9				204.7, C
10		218.7, C		208.7, C
11	2.90, m	43.4, CH	1.72, m	48.9, CH
12	1.07, d (6.4)	20.5, CH_3_	1.08, d (6.4)	16.7, CH_3_
13	1.41, d (6.4)	23.8, CH_3_	1.27, m; 1.63, m	27.6, CH_2_
14	0.88, s	18.6, CH_3_	0.77, t (7.4)	11.7, CH_3_
15	1.89, m; 1.34, m	35.4, CH_2_	3.16, dd (14.4, 7.4) 3.02, dd (14.4, 7.4)	22.3, CH_2_
16	2.10, m; 1.80, m	27.5, CH_2_	5.06, t (7.4)	121.3, CH
17	3.04, brs	54.7, CH		132.6, C
18		85.4, C	1.65, s	25.8, CH_3_
19	0.89, s	23.8, CH_3_	1.71, s	18.0, CH_3_
20	1.15, s	26.5, CH_3_	1.77, dd (13.0, 5.6) 2.67, dd (13.0, 11.0)	30.4, CH_2_
21	2.08, m; 1.70, m	30.9, CH_2_	4.55, dd (11.0, 5.6)	90.2, CH
22	5.31, brs	124.7, CH		71.0, C
23		133.4, C	1.39, s	27.1, CH_3_
24	1.62, s	18.1, CH_3_	1.22, s	24.2, CH_3_
25	1.78, s	26.1, CH_3_	1.52, m; 2.16, m	26.7, CH_2_
26	2.37, m	44.5, CH_2_	4.94, t (7.0)	122.4, CH
27	4.00, t (6.4)	76.1, CH		133.6, C
28		90.1, C	1.56, s	18.1, CH_3_
29	1.22, s	22.9, CH_3_	1.69, s	26.1, CH_3_
30	1.33, s	28.0, CH_3_	1.05, s	16.2, CH_3_
31	3.20, dd (14.4, 5.6)	36.9, CH	1.27, s	22.9, CH_3_
32	4.87, brs	120.4, CH		
33		136.0, C		
34	1.60, s	18.4, CH_3_		
35	1.68, s	26.2, CH_3_		
31-OH	2.66, brs			

^a^ Recorded in CD_3_OD (^1^H NMR 400 MHz, ^13^C NMR 125 MHz); ^b^ recorded in CDCl_3_ (^1^H NMR 400 MHz, ^13^C NMR 125 MHz).

**Table 2:** Hepatoprotective effects of compounds **1**–**11** (10 µm) against paracetamol-induced HepG2 cell ^a^.

Compound	OD Value	Cell Viability (% of Control)	Inhibition (% of Control)
Normal	1.717 ± 0.099	100.0	
Control	1.001 ± 0.041 ^***^	58.3	
GSH ^b^	1.328 ± 0.020 ^##^	77.3	45.7
1	1.091 ± 0.017 ^#^	63.6	12.6
2	0.982 ± 0.030	57.2	−2.7
3	1.028 ± 0.009	59.9	3.8
4	1.104 ± 0.053	64.3	14.4
5	1.033 ± 0.009	60.1	4.5
6	0.964 ± 0.010	56.1	−5.2
7	0.971 ± 0.002	56.6	−4.2
8	0.908 ± 0.037 ^#^	52.9	−13.0
9	0.935 ± 0.015	54.5	−9.2
10	0.817 ± 0.027 ^##^	47.6	−25.7
11	0.945 ± 0.016	55.1	−7.8

^a^ Results are expressed as the means ± SD (for samples, *n* = 3; for normal and control, *n* = 6; ^b^ positive control (20 µm); *** *p <* 0.01 vs. normal; ^#^ *p <* 0.05, ^##^ *p <* 0.01 vs. control.

## Data Availability

Data are contained within the article.

## Supplementary Materials

The following supporting information can be downloaded at: https://www.mdpi.com/article/10.3390/molecules27185932/s1, Figure S1: The HRESIMS spectrum of compound **1**, Figures S2 and S3: The UV/IR spectrum of compound **1**, Figures S4–S10: The 1D and 2D NMR spectra of compound **1** in CD_3_OD, Figure S11: The experimental ECD spectrum of compound **1**, Figure S12: The HRESIMS spectrum of compound **2**, Figures S13 and S14: The UV/IR spectrum of compound **2**, Figures S15–S19: The 1D and 2D NMR spectra of compound **2** in CDCl_3_, Figure S20: The experimental ECD spectrum of compound **2**, Figure S21: The flow chart for the separation of compounds **1**–**11**.

## Sample Availability

Samples of the compounds are not available from the authors.

## References

[1] Yang X.W., Grossman R., Xu G. (2018). Research progress of polycyclic polyprenylated acylphloroglucinols. Chem. Rev..

[2] Zhang L.J., Chiou C.T., Cheng J.J., Huang H.C., Kuo L.M.Y., Liao C.C., Bastow K.F., Lee K.H., Kuo Y.H. (2010). Cytotoxic polyisoprenyl benzophenonoids from *Garcinia subelliptica*. J. Nat. Prod..

[3] Guo Y., Zhang N., Chen C.M., Huang J.F., Li X.N., Liu J.J., Zhu H.C., Tong Q.Y., Zhang J.W., Luo Z.W. (2017). Tricyclic polyprenylated acylphloroglucinols from St John’s Wort, *Hypericum perforatum*. J. Nat. Prod..

[4] Li D.Y., Xue Y.B., Zhu H.C., Li Y., Sun B., Liu J.J., Yao G.M., Zhang J.W., Du G., Zhang Y.H. (2015). Hyperattenins A–I, bioactive polyprenylated acylphloroglucinols from *Hypericum attenuatum* Choisy. RSC Adv..

[5] Le D.H., Nishimura K., Takenaka Y., Mizushina Y., Tanahashi T. (2016). Polyprenylated benzoylphloroglucinols with DNA polymerase inhibitory activity from the fruits of *Garcinia schomburgkiana*. J. Nat. Prod..

[6] Chen J.J., Ting C.W., Hwang T.L., Chen I.S. (2009). Benzophenone derivatives from the fruits of *Garcinia multiflora* and their anti-inflammatory activity. J. Nat. Prod..

[7] Zhang J.S., Zou Y.H., Guo Y.Q., Li Z.Z., Tang G.H., Yin S. (2016). Polycyclic polyprenylated acylphloroglucinols: Natural phosphodiesterase-4 inhibitors from *Hypericum sampsonii*. RSC Adv..

[8] Lee J.Y., Duke R.K., Tran V.H., Hook J.M., Duke C.C. (2006). Hyperforin and its analogues inhibit CYP3A4 enzyme activity. Phytochemistry.

[9] Marti G., Eparvier V., Moretti C., Susplugas S., Prado S., Grellier P., Retailleau P., Gueritee F., Litaudon M. (2009). Antiplasmodial benzophenones from the trunk latex of *Moronobea coccinea* (Clusiaceae). Phytochemistry.

[10] Zhu H.C., Chen C.M., Yang J., Li X.N., Liu J.J., Sun B., Huang S.X., Li D.Y., Yao G.M., Luo Z.W. (2014). Bioactive acylphloroglucinols with adamantyl skeleton from *Hypericum sampsonii*. Org. Lett..

[11] Li Z.Q., Luo L., Ma G.Y., Chen X. (2004). Phloroglucinol and flavonoid constituents of *Hypericum acmosepaium*. J. Yunnan Univ..

[12] Wang X., Wang J.J., Suo X.Y., Sun H.R., Zhen B., Sun H., Li J.G., Ji T.F. (2020). Hyperacmosins H–J, three new polycyclic polyprenylated acylphloroglucinol derivatives from *Hypericum acmosepalum*. J. Asian Nat. Prod. Res..

[13] Wang X., Shi M.J., Wang J.J., Suo X.Y., Sun H.R., Zhen B., Sun H., Li J.G., Ji T.F. (2020). Hyperacmosins E–G, three new homoadamantane-type polyprenylated acylphloroglucinols from *Hypericum acmosepalum*. Fitoterapia.

[14] Suo X.Y., Shi M.J., Dang J., Yue H.L., Tao Y.D., Zhen B., Wang J.J., Wang X., Sun H.R., Sun H. (2021). Two new polycyclic polyprenylated acylphloroglucinols derivatives from *Hypericum acmosepalum*. J. Asian Nat. Prod. Res..

[15] Sun M.X., Dang J., Zhu T.T., Wang X., Suo X.Y., Wang J.J., Ji T.F., Liu B. (2021). Hyperacmosin N, new acylphloroglucinol derivative with complicated caged core from *Hypericum acmosepalum*. Tetrahedron.

[16] Sun M.X., Wang X., Zhu T.T., Suo X.Y., Wang J.J., Ji T.F., Liu B. (2021). Hyperacmosins K–M, three new polycyclic polyprenylated acylphloroglucinols from *Hypericum acmosepalum*. RSC Adv..

[17] Fukuyama Y., Minami H., Kuwayama A. (1998). Garsubellins, polyisoprenylated phloroglucinol derivatives from *Garcinia subelliptica*. Phytochemistry.

[18] Hashida C., Tanaka N., Kashiwada Y., Ogawa M., Takaishi Y. (2008). Prenylated phloroglucinol derivatives from *Hypericum perforatum* var. Angustifolium. Chem. Pharm. Bull..

[19] Yang J.B., Liu R.D., Ren J., Wei Q., Wang A.G., Su Y.L. (2016). Two new prenylated phloroglucinol derivatives from *Hypericum scabrum*. J. Asian Nat. Prod. Res..

[20] Liu X., Yang X.W., Chen C.Q., Wu C.Y., Zhang J.J., Ma J.Z., Wang H., Yang L.X., Xu G. (2013). Bioactive polyprenylated acylphloroglucinol derivatives from *Hypericum cohaerens*. J. Nat. Prod..

[21] Ma J., Xia G.Y., Zang Y.D., Li C.J., Yang J.B., Huang J.W., Zhang J.J., Su Y.L., Wang A.G., Zhang D.M. (2021). Three new decarbonyl prenylphloroglucinols bearing unusual spirost subunits from *Hypericum scabrum* and their neuronal activities. Chinese Chem. Lett..

