# Supplementary material for: Hyperacmosin R, a New Decarbonyl Prenylphloroglucinol with Unusual Spiroketal Subunit from Hypericum acmosepalum
Source: Molecules. 2022 Sep 13;27(18):5932. doi: 10.3390/molecules27185932 (PMC9501122; doi:10.3390/molecules27185932)

# Hyperacmosin R, a New Decarbonyl Prenylphloroglucinol with Unusual Spiroketal Subunit from *Hypericum acmosepalum*

**Yonghui Ma [1,2,†], Xiaoyu Liu [2,†], Bo Liu [3], Pingping Li [2], Xinyue Suo [2], Tingting Zhu [2], Tengfei Ji [2,4,*], Jin Li [1,*] and Xiaoxiu Li [3,*]**

[1] The Key Laboratory of Plant Stress Biology in Arid Land, College of Life Sciences, Xinjiang Normal University, Urumqi 830054, China
[2] State Key Laboratory of Bioactive Substance and Function of Natural Medicines, Institute of Materia Medica, Chinese Academy of Medical Sciences and Peking Union Medical College, Beijing 100050, China
[3] School of Pharmacy, Shenyang Medical College, Shenyang 110034, China
[4] Key Laboratory of Tibetan Medicine Research, Northwest Institute of Plateau Biology, Chinese Academy of Sciences, Xining 810008, China
* Correspondence: jitf@imm.ac.cn (T.J.); xjcjlj4@xjnu.edu.cn (J.L.); sylixiaoxiu@163.com (X.L.)
† These authors contributed equally to this work.

# Supporting Information

## CONTENTS

Physical and Spectroscopic Data of Compounds **3**–**11**.

**Figure S1.** The HRESIMS spectrum of Hyperacmosin R (**1**).

**Figure S2.** The UV spectrum of Hyperacmosin R (**1**).

**Figure S3.** The IR spectrum of Hyperacmosin R (**1**).

**Figure S4.** The $^{1}H$ NMR spectrum of Hyperacmosin R (**1**) in $CD_3OD$ (400 MHz).

**Figure S5.** The enlarged $^{1}H$ NMR spectrum of Hyperacmosin R (**1**) in $CD_3OD$ (400 MHz)

**Figure S6.** The $^{13}C$ NMR spectrum of Hyperacmosin R (**1**) in $CD_3OD$ (125 MHz).

**Figure S7.** The $^{1}H$-$^{1}H$ COSY spectrum of Hyperacmosin R (**1**).

**Figure S8.** The HSQC spectrum of Hyperacmosin R (**1**).

**Figure S9.** The HMBC spectrum of Hyperacmosin R (**1**).

**Figure S10.** The ROESY spectrum of Hyperacmosin R (**1**).

**Figure S11.** The experimental ECD spectrum of Hyperacmosin R (**1**).

**Figure S12.** The HRESIMS spectrum of Hyperacmosin S (**2**).

**Figure S13.** The UV spectrum of Hyperacmosin S (**2**).

**Figure S14.** The IR spectrum of Hyperacmosin S (**2**).

**Figure S15.** The $^{1}H$ NMR spectrum of Hyperacmosin S (**2**) in $CDCl_3$ (400 MHz).

**Figure S16.** The $^{13}C$ NMR spectrum of Hyperacmosin S (**2**) in $CDCl_3$ (125 MHz).

**Figure S17.** The HSQC spectrum of Hyperacmosin S (**2**).

**Figure S18.** The HMBC spectrum of Hyperacmosin S (**2**).

**Figure S19.** The ROESY spectrum of Hyperacmosin S (**2**).

**Figure S20.** The experimental ECD spectrum of Hyperacmosin S (**2**).

**Figure S21.** The flow chart for the separation of compounds **1-11**.

Physical and Spectroscopic Data of Compounds **3**–**11**.

Furoadhyperforin isomer A (**3**):

colorless oil; $[\alpha]^{20}_{D}$ +81.2 (c 0.10, MeOH); $^1$H NMR (400 MHz, $CDCl_3$, $\delta$ ppm, $J$ Hz) $\delta$: 1.46 (m, 1H, H-6a), 1.85 (m, 1H, H-6b), 1.55 (m, 1H, H-7), 1.93 (m, 1H, H-11), 1.11 (d, 3H, $J$ = 6.4 Hz, H-12), 2.10 (m, 2H, H-13), 0.81 (t, 3H, $J$ = 7.4 Hz, H-14), 2.86 (dd, 1H, $J$ = 15.0, 8.2 Hz, H-15a), 2.98 (dd, 1H, $J$ = 15.0, 10.4 Hz, H-15b), 4.74 (dd, 1H, $J$ = 10.4, 8.2 Hz, H-16), 1.27 (s, 3H, H-18), 1.21 (s, 3H, H-19), 2.51 (dd, 1H, $J$ = 14.4, 6.8 Hz, H-20a), 2.42 (dd, 1H, $J$ = 14.4, 7.8 Hz, H-20b), 5.10 (m, 1H, H-21), 1.66 (s, 3H, H-23), 1.69 (s, 3H, H-24), 1.70 (m, 1H, H-25a), 2.10 (m, 1H, H-25b), 4.92 (t, 1H, $J$ = 7.4 Hz, H-26), 1.55 (s, 3H, H-28), 1.66 (s, 3H, H-29), 1.02 (s, 3H, H-30), 1.46 (m, 1H, H-31a), 1.90 (m, 1H, H-31b), 1.90 (m, 1H, H-32a), 2.10 (m, 1H, H-32b), 5.05 (m, 1H, H-33), 1.59 (s, 3H, H-35), 1.64 (s, 3H, H-36). $^{13}$C NMR (125 MHz, $CDCl_3$, $\delta$ ppm) $\delta$: 83.5 (C-1), 187.4 (C-2), 118.2 (C-3), 176.5 (C-4), 54.5 (C-5), 37.6 (C-6), 43.2 (C-7), 48.3 (C-8), 205.9 (C-9), 209.5 (C-10), 48.7 (C-11), 16.4 (C-12), 27.3 (C-13), 11.5 (C-14), 27.1 (C-15), 92.3 (C-16), 71.9 (C-17), 23.5 (C-18), 24.8 (C-19), 28.9 (C-20), 118.3 (C-21), 134.8 (C-22), 24.9 (C-23), 17.6 (C-24), 27.0 (C-25), 122.4 (C-26), 133.5 (C-27), 25.9 (C-28), 17.9 (C-29), 13.5 (C-30), 36.6 (C-31), 25.6 (C-32), 124.6 (C-33), 131.1 (C-34), 25.7 (C-35), 17.7 (C-36). ESIMS ($m/z$): 567 $[M + H]^+$.

Furoadhyperforin isomer B (**4**):

colorless oil; $[\alpha]^{20}_{D}$ +17.8 (c 0.18, MeOH); $^1$H NMR (400 MHz, $CDCl_3$, $\delta$ ppm, $J$ Hz) $\delta$: 1.44 (m, 1H, H-6a), 1.90 (m, 1H, H-6b), 1.65 (m, 1H, H-7), 1.88 (m, 1H, H-11), 1.12 (d, 3H, $J$ = 6.4 Hz, H-12), 2.10 (m, 2H, H-13), 0.82 (t, 3H, $J$ = 7.4 Hz, H-14), 2.99 (dd, 1H, $J$ = 14.6, 10.0 Hz, H-15a), 2.93 (dd, 1H, $J$ = 14.6, 8.0 Hz, H-15b), 4.79 (dd, 1H, $J$ = 10.0, 8.0 Hz, H-16), 1.30 (s, 3H, H-18), 1.17 (s, 3H, H-19), 2.47 (m, 2H, H-20), 5.00 (m, 1H, H-21), 1.69 (s, 3H, H-23), 1.69 (s, 3H, H-24), 1.80 (m, 1H, H-25a), 2.12 (m, 1H, H-25b), 4.95 (m, 1H, H-26), 1.57 (s, 3H, H-28), 1.70 (s, 3H, H-29), 1.02 (s, 3H, H-30), 1.48 (m, 1H, H-31a), 1.89 (m, 1H, H-31b), 1.86 (m, 1H, H-32a), 2.08 (m, 1H, H-32b), 5.05 (m, 1H, H-33), 1.59 (s, 3H, H-35), 1.64 (s, 3H, H-36). $^{13}$C NMR (125 MHz, $CDCl_3$, $\delta$ ppm) $\delta$: 83.5 (C-1), 187.1 (C-2), 119.0 (C-3), 176.1 (C-4), 54.4 (C-5), 38.4 (C-6), 42.7 (C-7), 48.4 (C-8), 206.2 (C-9), 208.9 (C-10), 48.8 (C-11), 16.4 (C-12), 27.3 (C-13), 11.4 (C-14), 27.2 (C-15), 93.0 (C-16), 71.5 (C-17), 23.0 (C-18), 26.2 (C-19), 29.2 (C-20), 120.0 (C-21), 134.6 (C-22), 25.6 (C-23), 17.6 (C-24), 26.8 (C-25), 122.3 (C-26), 133.4 (C-27), 24.7 (C-28), 18.1 (C-29), 13.8 (C-30), 36.5 (C-31), 25.7 (C-32), 124.5 (C-33), 131.1 (C-34), 25.8 (C-35), 17.9 (C-36). ESIMS ($m/z$): 567 $[M + H]^+$.

Furohyperforin isomer 2a (**5**):

colorless oil; $[\alpha]^{20}_{D}$ +98.9 (c 0.09, MeOH); $^1$H NMR (400 MHz, $CDCl_3$, $\delta$ ppm, $J$ Hz) $\delta$: 1.42 (m, 1H, H-6a), 1.82 (dd, 1H, $J$ = 13.6, 4.2 Hz, H-6b), 1.67 (m, 1H, H-7), 2.28 (m, 1H, H-11), 1.12 (d, 3H, $J$ = 6.4 Hz, H-12), 1.67 (m, 1H, H-13a), 2.07 (m, 1H, H-13b), 0.89 (t, 3H, $J$ = 7.6 Hz, H-14), 2.99 (dd, 2H, $J$ = 10.6, 2.4 Hz, H-15), 4.75 (t, 1H, $J$ = 10.4 Hz, H-16), 1.38 (s, 3H, H-18), 1.24 (s, 3H, H-19), 2.43 (m, 2H, H-20), 5.00 (m, 1H, H-21), 1.65 (s, 3H, H-23), 1.65 (s, 3H, H-24), 1.47 (m, 1H, H-25a), 1.77 (m, 1H, H-25b), 4.93 (m, 1H, H-26), 1.57 (s, 3H, H-28), 1.65 (s, 3H, H-29), 1.12 (s, 3H, H-30), 1.61 (m, 2H, H-31), 1.97 (m, 2H, H-32), 4.98 (m, 1H, H-33), 1.54 (s, 3H, H-35), 1.67 (s, 3H, H-36). $^{13}$C NMR (125 MHz, $CDCl_3$, $\delta$ ppm) $\delta$: 73.9 (C-1), 171.5 (C-2), 120.6 (C-3), 190.6 (C-4), 63.9 (C-5), 39.5 (C-6), 42.5 (C-7), 47.7 (C-8), 205.6 (C-9), 208.2 (C-10), 47.6 (C-11), 16.7 (C-12), 28.1 (C-13), 11.2 (C-14), 26.6 (C-15), 93.9 (C-16), 70.9 (C-17), 26.7 (C-18), 25.4 (C-19), 29.2 (C-20), 119.5 (C-21), 134.1 (C-22), 25.9 (C-23), 18.0 (C-24), 26.5 (C-25), 122.3 (C-26), 133.4 (C-27), 25.9 (C-28), 17.9 (C-29), 14.3 (C-30), 38.2 (C-31), 24.4 (C-32), 124.4 (C-33), 131.6 (C-34), 25.6 (C-35), 17.7 (C-36). ESIMS ($m/z$): 567 $[M + H]^+$.

Furohyperforin isomer 2b (**6**):

colorless oil; $[\alpha]^{20}_{D}$ +5.1 (c 0.16, MeOH); $^1$H NMR (400 MHz, $CDCl_3$, $\delta$ ppm, $J$ Hz) $\delta$: 1.37 (m, 1H, H-6a), 1.81 (m, 1H, H-6b), 1.55 (m, 1H, H-7), 2.15 (m, 1H, H-11), 1.16 (d, 3H, $J$ = 6.4 Hz, H-12), 1.25 (m, 1H, H-13a), 1.77 (m, 1H, H-13b), 0.88 (t, 3H, $J$ = 7.6 Hz, H-14), 2.98 (dd, 1H, $J$ = 15.0,

10.4 Hz, H-15a), 3.05 (dd, 1H, *J* = 15.0, 10.4 Hz, H-15b), 4.62 (t, 1H, *J* = 10.4 Hz, H-16), 1.33 (s, 3H, H-18), 1.24 (s, 3H, H-19), 2.43 (m, 2H, H-20), 5.00 (dd, 1H, *J* = 16.2, 7.2 Hz, H-21), 1.66 (s, 3H, H-23), 1.66 (s, 3H, H-24), 1.70 (m, 1H, H-25a), 2.07 (m, 1H, H-25b), 4.91 (m, 1H, *J* = 7.2 Hz, H-26), 1.52 (s, 3H, H-28), 1.66 (s, 3H, H-29), 1.11 (s, 3H, H-30), 1.43 (m, 1H, H-31a), 1.81 (m, 1H, H-31b), 2.00 (m, 1H, H-32a), 2.16 (m, 1H, H-32b), 5.00 (dd, 1H, *J* = 16.2, 7.2 Hz, H-33), 1.58 (s, 3H, H-35), 1.66 (s, 3H, H-36). $^{13}$C NMR (125 MHz, $CDCl_3$, $\delta$ ppm) $\delta$: 74.6 (C-1), 171.7 (C-2), 120.3 (C-3), 190.8 (C-4), 63.6 (C-5), 39.5 (C-6), 44.2 (C-7), 47.2 (C-8), 205.7 (C-9), 208.4 (C-10), 47.6 (C-11), 17.0 (C-12), 27.9 (C-13), 11.8 (C-14), 26.7 (C-15), 93.5 (C-16), 71.0 (C-17), 27.0 (C-18), 25.1 (C-19), 29.1 (C-20), 119.5 (C-21), 134.2 (C-22), 25.9 (C-23), 18.1 (C-24), 26.4 (C-25), 122.3 (C-26), 133.4 (C-27), 25.8 (C-28), 17.9 (C-29), 12.8 (C-30), 39.2 (C-31), 24.8 (C-32), 124.3 (C-33), 132.2 (C-34), 25.6 (C-35), 17.8 (C-36). ESIMS (*m*/*z*): 567 $[M + H]^+$.

Furohyperforin isomer 2 (**7**):

colorless oil; $[\alpha]^{20}_D$ -19.3 (c 0.33, MeOH); $^1$H NMR (400 MHz, $CDCl_3$, $\delta$ ppm, *J* Hz) $\delta$: 1.43 (m, 1H, H-6a), 1.92 (dd, 1H, *J* = 13.6, 4.4 Hz, H-6b), 1.67 (m, 1H, H-7), 2.12 (m, 1H, H-11), 1.13 (d, 3H, *J* = 6.6 Hz, H-12), 1.05 (d, 3H, *J* = 6.6 Hz, H-13), 2.93 (dd, 1H, *J* = 12.2, 5.8 Hz, H-14a), 2.99 (dd, 1H, *J* = 12.2, 7.4 Hz, H-14b), 4.80 (dd, 1H, *J* = 7.4, 5.8 Hz, H-15), 1.30 (s, 3H, H-17), 1.17 (s, 3H, H-18), 2.45 (dd, 1H, *J* = 15.2, 9.0 Hz, H-19a), 2.52 (d, 1H, *J* = 15.2 Hz, H-19b), 5.00 (m, 1H, H-20), 1.69 (s, 3H, H-22), 1.69 (s, 3H, H-23), 1.54 (m, 1H, H-24a), 1.86 (m, 1H, H-24b), 5.05 (m, 1H, H-25), 1.59 (s, 3H, H-27), 1.65 (s, 3H, H-28), 1.05 (s, 3H, H-29), 1.44 (m, 2H, H-30), 1.80 (m, 1H, H-31a), 2.11 (m, 1H, H-31b), 4.96 (t, 1H, *J* = 7.1 Hz, H-32), 1.59 (s, 3H, H-34), 1.70 (s, 3H, H-35). $^{13}$C NMR (125 MHz, $CDCl_3$, $\delta$ ppm) $\delta$: 84.2 (C-1), 187.0 (C-2), 119.2 (C-3), 176.2 (C-4), 54.8 (C-5), 37.5 (C-6), 42.9 (C-7), 48.8 (C-8), 206.4 (C-9), 209.8 (C-10), 42.7 (C-11), 20.9 (C-12), 21.9 (C-13), 26.0 (C-14), 92.3 (C-15), 72.2 (C-16), 25.5 (C-17), 22.2 (C-18), 28.3 (C-19), 119.1 (C-20), 135.2 (C-21), 24.8 (C-22), 17.3 (C-23), 35.6 (C-24), 124.9 (C-25), 131.3 (C-26), 24.8 (C-27), 16.8 (C-28), 13.0 (C-29), 36.9 (C-30), 26.9 (C-31), 122.7 (C-32), 133.8 (C-33), 25.0 (C-34), 17.0 (C-35). ESIMS (*m*/*z*): 553 $[M + H]^+$.

Furohyperforin (**8**):

colorless oil; $[\alpha]^{20}_D$ +68.7 (c 0.31, MeOH); $^1$H NMR (400 MHz, $CDCl_3$, $\delta$ ppm, *J* Hz) $\delta$: 1.51(t, 1H, *J* = 12.8 Hz, H-6a), 2.01 (m, 1H, H-6b), 1.62 (m, 1H, H-7), 1.99 (m, 1H, H-11), 1.09 (d, 3H, *J* = 5.8 Hz, H-12), 1.00 (d, 3H, *J* = 5.8 Hz, H-13), 3.01 (dd, 1H, *J* = 13.6, 7.6 Hz, H-14a), 3.14 (dd, 1H, *J* = 13.6, 6.4 Hz, H-14b), 5.07 (dd, 1H, *J* = 5.0 Hz, H-15), 1.64 (s, 3H, H-17), 1.69 (s, 3H, H-18), 1.78 (t, 1H, *J* = 7.2 Hz, H-19a), 2.65 (t, 1H, *J* = 11.8 Hz, H-19b), 4.55 (dd, 1H, *J* = 9.8, 5.2 Hz, H-20), 1.38 (s, 3H, H-22), 1.21 (s, 3H, H-23), 1.77 (m, 1H, H-24a), 2.17 (m, 1H, H-24b), 4.94 (m, 1H, H-25), 1.69 (s, 3H, H-27), 1.57 (s, 3H, H-28), 1.04 (s, 3H, H-29), 1.32 (m, 1H, H-30a), 2.06 (m, 1H, H-30b), 1.93 (m, 1H, H-31a), 2.15 (m, 1H, H-31b), 5.07 (d, 1H, *J* = 5.0 Hz, H-32), 1.60 (s, 3H, H-34), 1.64 (s, 3H, H-35). $^{13}$C NMR (125 MHz, $CDCl_3$, $\delta$ ppm) $\delta$: 83.2 (C-1), 192.7 (C-2), 116.6 (C-3), 172.9 (C-4), 59.4 (C-5), 38.0 (C-6), 43.3 (C-7), 48.2 (C-8), 204.5 (C-9), 209.5 (C-10), 42.0 (C-11), 20.4 (C-12), 21.4 (C-13), 22.1 (C-14), 121.2 (C-15), 132.5 (C-16), 25.6 (C-17), 17.8 (C-18), 30.2 (C-19), 90.1 (C-20), 70.8 (C-21), 26.9 (C-22), 24.0 (C-23), 27.1 (C-24), 122.3 (C-25), 133.5 (C-26), 25.9 (C-27), 18.0 (C-28), 13.4 (C-29), 36.3 (C-30), 25.2 (C-31), 124.7 (C-32), 131.1 (C-33), 25.7 (C-34), 17.7 (C-35). ESIMS (*m*/*z*): 553 $[M + H]^+$.

Furoadhyperforin (**9**):

colorless oil; $[\alpha]^{20}_D$ +77.6 (c 0.22, MeOH); $^1$H NMR (400 MHz, $CDCl_3$, $\delta$ ppm, *J* Hz) $\delta$: 1.51(t, 1H, *J* = 12.8 Hz, H-6a), 2.01 (dd, 1H, *J* = 13.0, 4.0 Hz, H-6b), 1.62 (m, 1H, H-7), 1.74 (m, 1H, H-11), 1.09 (d, 3H, *J* = 6.6 Hz, H-12), 1.27 (m, 1H, H-13a), 1.69 (m, 1H, H-13b), 0.77 (t, 3H, *J* = 7.4 Hz, H-14), 3.01 (dd, 1H, *J* = 14.4, 7.6 Hz, H-15a), 3.15 (dd, 1H, *J* = 14.4, 7.0 Hz, H-15b), 5.06 (m, 1H, H-16), 1.64 (s, 3H, H-18), 1.70 (s, 3H, H-19), 1.76 (dd, 1H, *J* = 13.0, 5.6 Hz, H-20a), 2.66 (dd, 1H, *J* = 13.0, 10.8 Hz, H-20b), 4.54 (dd, 1H, *J* = 10.8, 5.6 Hz, H-21), 1.38 (s, 3H, H-23), 1.22 (s, 3H, H-24), 1.77 (m, 1H, H-25a), 2.17 (m, 1H, H-25b), 4.94 (t, 1H, *J* = 7.0 Hz, H-26), 1.70 (s, 3H, H-28),

1.57 (s, 3H, H-29), 1.05 (s, 3H, H-30), 1.32 (m, 1H, H-31a), 2.07 (m, 1H, H-31b), 1.92 (m, 1H, H-32a), 2.16 (m, 1H, H-32b), 5.06 (d, 1H, *J* = 7.4 Hz, H-33), 1.59 (s, 3H, H-35), 1.64 (s, 3H, H-36). $^{13}$C NMR (125 MHz, $CDCl_3$, *δ* ppm) *δ*: 83.3 (C-1), 192.8 (C-2), 116.6 (C-3), 172.9 (C-4), 59.3 (C-5), 38.0 (C-6), 43.3 (C-7), 48.3 (C-8), 204.5 (C-9), 209.0 (C-10), 48.7 (C-11), 16.6 (C-12), 27.4 (C-13), 11.5 (C-14), 22.2 (C-15), 121.2 (C-16), 132.4 (C-17), 25.7 (C-18), 17.8 (C-19), 30.2 (C-20), 90.0 (C-21), 70.8 (C-22), 26.9 (C-23), 24.0 (C-24), 27.1 (C-25), 122.3 (C-26), 133.5 (C-27), 25.9 (C-28), 18.0 (C-29), 13.4 (C-30), 36.4 (C-31), 25.3 (C-32), 124.7 (C-33), 131.1 (C-34), 25.6 (C-35), 17.7 (C-36). ESIMS (*m*/*z*): 567 $[M + H]^+$.

Hypercohin E (**10**):

colorless oil; $[\alpha]^{20}_D$ +44.3 (c 0.12, MeOH); $^1$H NMR (400 MHz, $CDCl_3$, *δ* ppm, *J* Hz) *δ*: 1.46(m, 1H, H-6a), 2.33 (m, 1H, H-6b), 1.50 (m, 1H, H-7), 1.98 (m, 1H, H-11), 1.07 (d, 3H, *J* = 6.4 Hz, H-12), 1.00 (d, 3H, *J* = 6.4 Hz, H-13), 3.11 (dd, 1H, *J* = 14.2, 7.6 Hz, H-14a), 3.05 (m, 1H, H-14b), 5.11 (t, 1H, *J* = 8.0 Hz, H-15), 1.68 (s, 3H, H-17), 1.64 (s, 3H, H-18), 1.79 (dd, 1H, *J* = 13.6, 10.2 Hz, H-19a), 3.01 (dd, 1H, *J* = 13.6, 5.2 Hz, H-19b), 4.35 (dd, 1H, *J* = 10.2, 5.2 Hz, H-20), 1.42 (s, 3H, H-22), 1.22 (s, 3H, H-23), 1.72 (m, 1H, H-24a), 2.13 (m, 1H, H-24b), 4.94 (t, 1H, *J* = 7.2 Hz, H-25), 1.54 (s, 3H, H-27), 1.65 (s, 3H, H-28), 1.03 (s, 3H, H-29), 1.26 (m, 1H, H-30a), 2.08 (m, 1H, H-30b), 1.94 (m, 1H, H-31a), 2.16 (m, 1H, H-31b), 5.07 (d, 1H, *J* = 7.6 Hz, H-32), 1.60 (s, 3H, H-34), 1.64 (s, 3H, H-35). $^{13}$C NMR (125 MHz, $CDCl_3$, *δ* ppm) *δ*: 83.1 (C-1), 193.1 (C-2), 117.8 (C-3), 173.0 (C-4), 59.3 (C-5), 40.7 (C-6), 44.7 (C-7), 47.6 (C-8), 204.4 (C-9), 209.9 (C-10), 41.9 (C-11), 21.5 (C-12), 20.5 (C-13), 22.7 (C-14), 121.2 (C-15), 132.7 (C-16), 25.8 (C-17), 17.8 (C-18), 28.7 (C-19), 91.4 (C-20), 71.0 (C-21), 27.2 (C-22), 25.5 (C-23), 27.1 (C-24), 122.4 (C-25), 133.4 (C-26), 25.9 (C-27), 17.9 (C-28), 13.4 (C-29), 36.4 (C-30), 25.5 (C-31), 125.0 (C-32), 131.2 (C-33), 25.9 (C-34), 18.0 (C-35). ESIMS (*m*/*z*): 553 $[M + H]^+$.

Hypercohin F (**11**):

colorless oil; $[\alpha]^{20}_D$ +72.1 (c 0.06, MeOH); $^1$H NMR (400 MHz, $CDCl_3$, *δ* ppm, *J* Hz) *δ*: 1.47 (m, 1H, H-6a), 2.33 (m, 1H, H-6b), 1.50 (m, 1H, H-7), 1.77 (m, 1H, H-11), 1.08 (d, 3H, *J* = 6.4 Hz, H-12), 1.26 (m, 1H, H-13a), 1.70 (m, 1H, H-13b), 0.77 (t, 3H, *J* = 7.4 Hz, H-14), 3.05 (m, 1H, H-15a), 3.12 (dd, 1H, *J* = 14.0, 7.4 Hz, H-15b), 5.11 (t, 1H, *J* = 7.4 Hz, H-16), 1.64 (s, 3H, H-18), 1.69 (s, 3H, H-19), 1.79 (dd, 1H, *J* = 10.0, 9.6 Hz, H-20a), 3.01 (dd, 1H, *J* = 9.6, 5.2 Hz, H-20b), 4.36 (dd, 1H, *J* = 10.0, 5.2 Hz, H-21), 1.42 (s, 3H, H-23), 1.22 (s, 3H, H-24), 1.74 (m, 1H, H-25a), 2.13 (m, 1H, H-25b), 4.94 (t, 1H, *J* = 7.2 Hz, H-26), 1.66 (s, 3H, H-28), 1.55 (s, 3H, H-29), 1.04 (s, 3H, H-30), 1.25 (m, 1H, H-31a), 2.08 (m, 1H, H-31b), 1.94 (m, 1H, H-32a), 2.17 (m, 1H, H-32b), 5.07 (d, 1H, *J* = 7.6 Hz, H-33), 1.60 (s, 3H, H-35), 1.64 (s, 3H, H-36). $^{13}$C NMR (125 MHz, $CDCl_3$, *δ* ppm) *δ*: 83.2 (C-1), 193.2 (C-2), 117.8 (C-3), 173.1 (C-4), 59.3 (C-5), 40.8 (C-6), 44.7 (C-7), 47.7 (C-8), 204.5 (C-9), 209.3 (C-10), 48.7 (C-11), 16.7 (C-12), 27.5 (C-13), 11.7 (C-14), 22.8 (C-15), 121.2 (C-16), 132.6 (C-17), 25.9 (C-18), 17.8 (C-19), 28.7 (C-20), 91.4 (C-21), 71.1 (C-22), 27.3 (C-23), 25.5 (C-24), 27.1 (C-25), 122.4 (C-26), 133.4 (C-27), 26.0 (C-28), 18.1 (C-29), 13.3 (C-30), 36.5 (C-31), 25.6 (C-32), 124.9 (C-33), 131.2 (C-34), 25.9 (C-35), 17.9 (C-36). ESIMS (*m*/*z*): 567 $[M + H]^+$.

**Figure S1.** The HRESIMS spectrum of Hyperacmosin R (**1**).

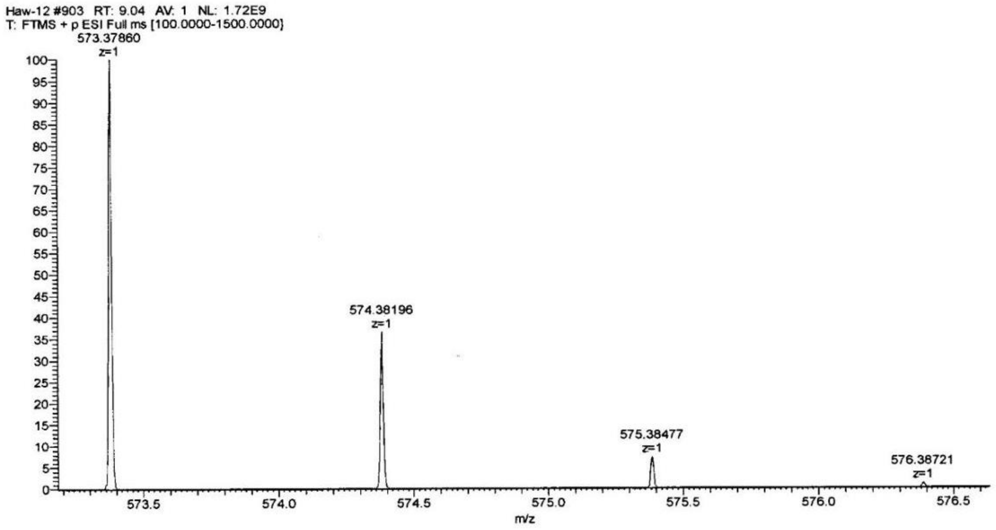


| m/z | Theo. Mass | Delta (ppm) | RDB equiv. | Composition | |
|---|---|---|---|---|---|
| | | | | | |
| 573.37860 | 573.37858 | 0.03 | 8.5 | C34 H53 O7 | M+H |

**Figure S2.** The UV spectrum of Hyperacmosin R (**1**).

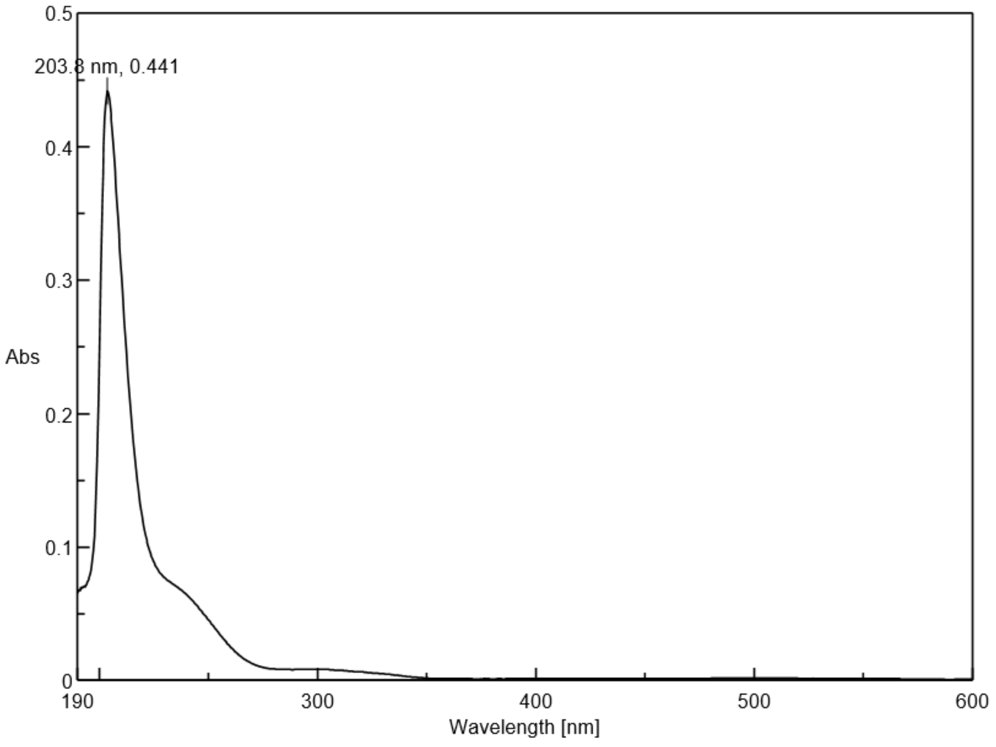

**Figure S3.** The IR spectrum of Hyperacmosin R (**1**).

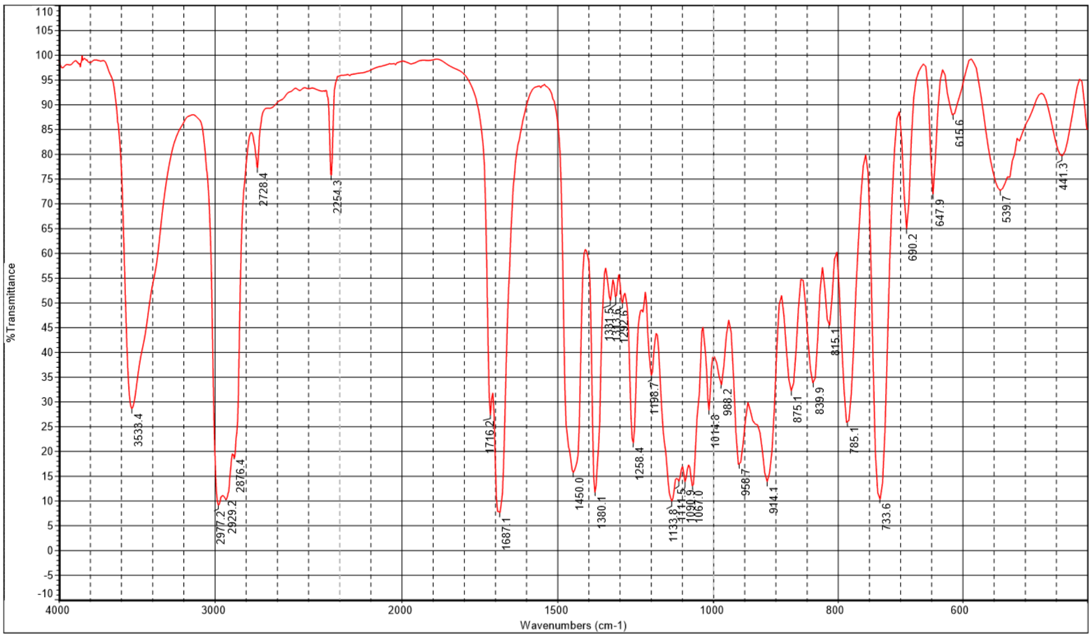


**Figure S4.** The $^{1}H$ NMR spectrum of Hyperacmosin R (**1**) in $CD_3OD$ (400 MHz).

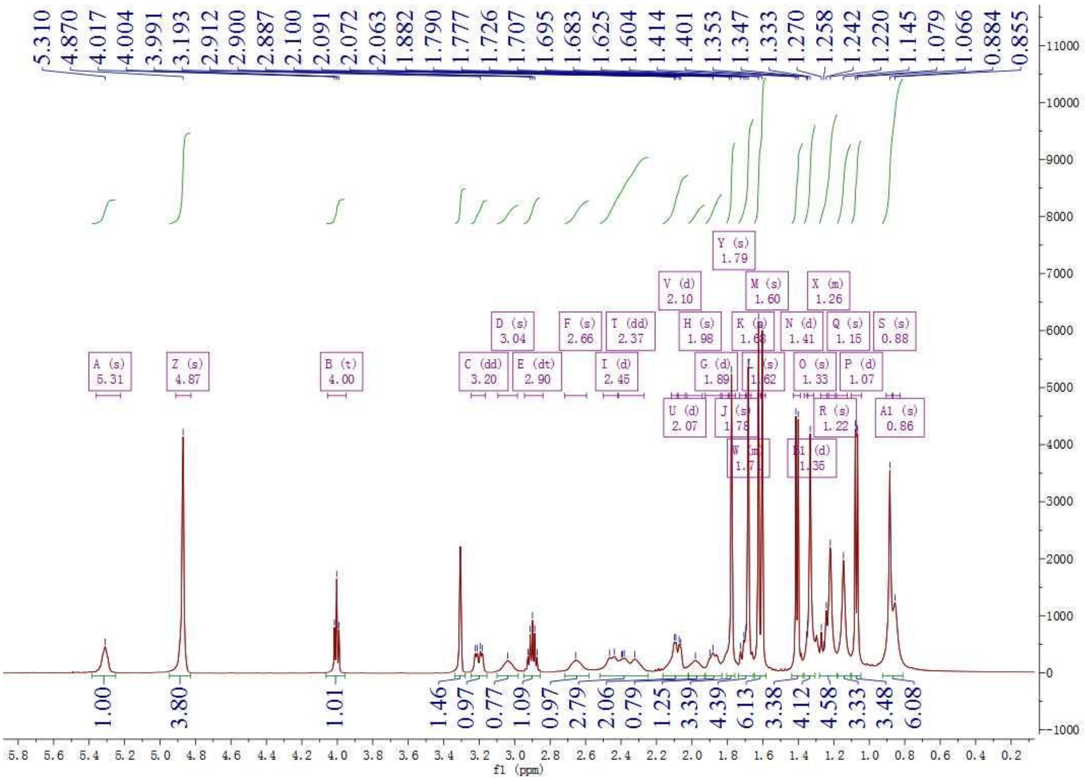

**Figure S5.** The enlarged $^{1}H$ NMR spectrum of Hyperacmosin R (**1**) in $CD_3OD$ (400 MHz).

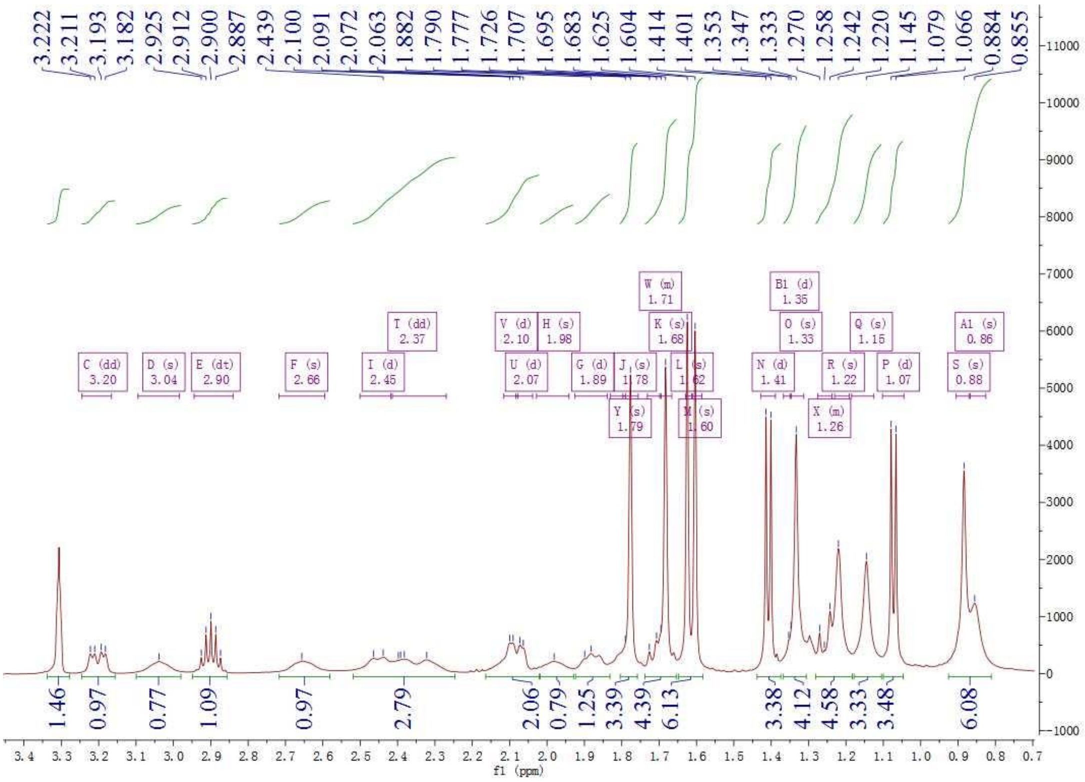


**Figure S6.** The $^{13}C$ NMR spectrum of Hyperacmosin R (**1**) in $CD_3OD$ (125 MHz).

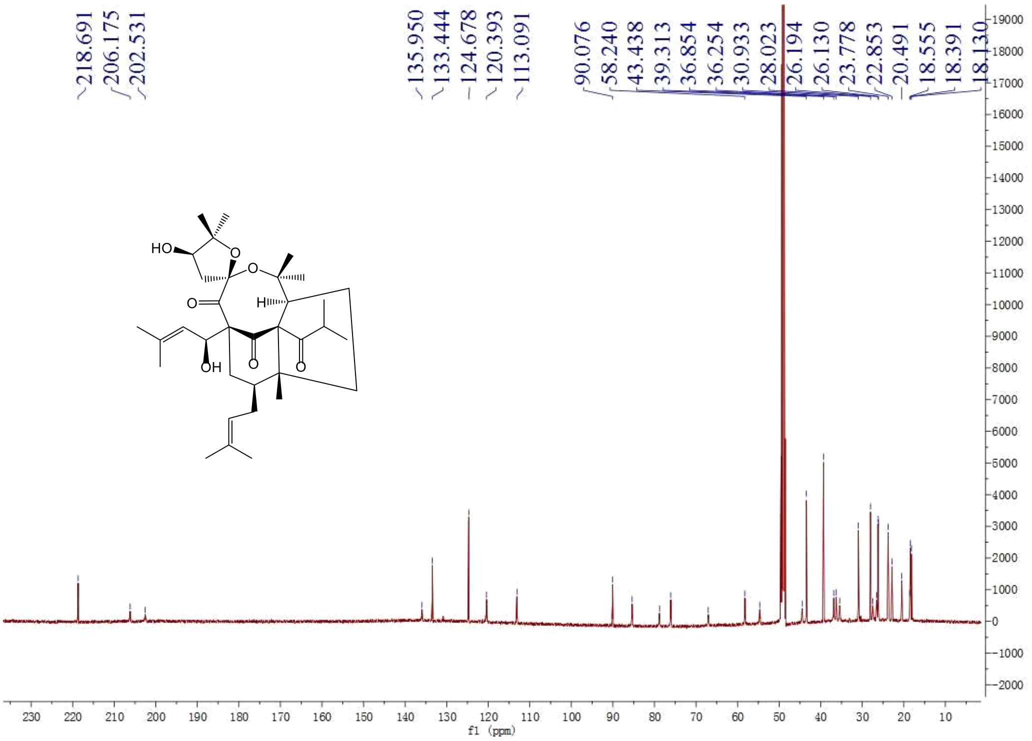

**Figure S7.** $^{1}H$-$^{1}H$ COSY spectrum of Hyperacmosin R (**1**).

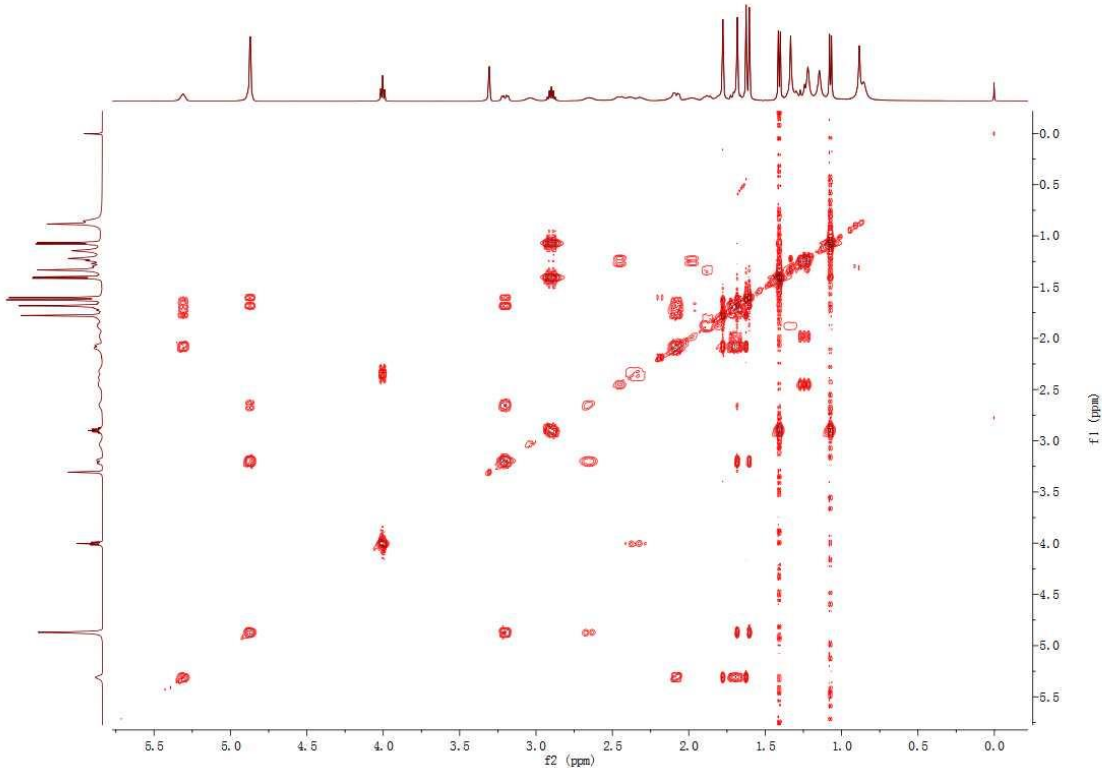


**Figure S8.** The HSQC spectrum of Hyperacmosin R (**1**).

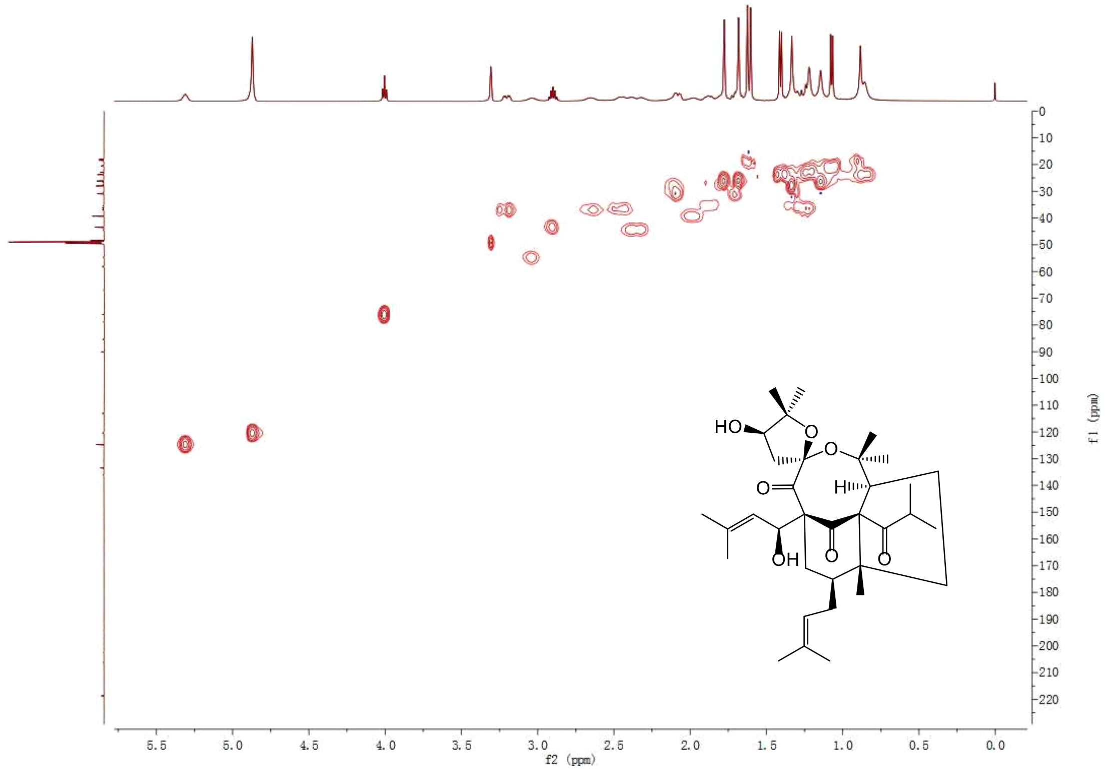

**Figure S9.** The HMBC spectrum of Hyperacmosin R (**1**).

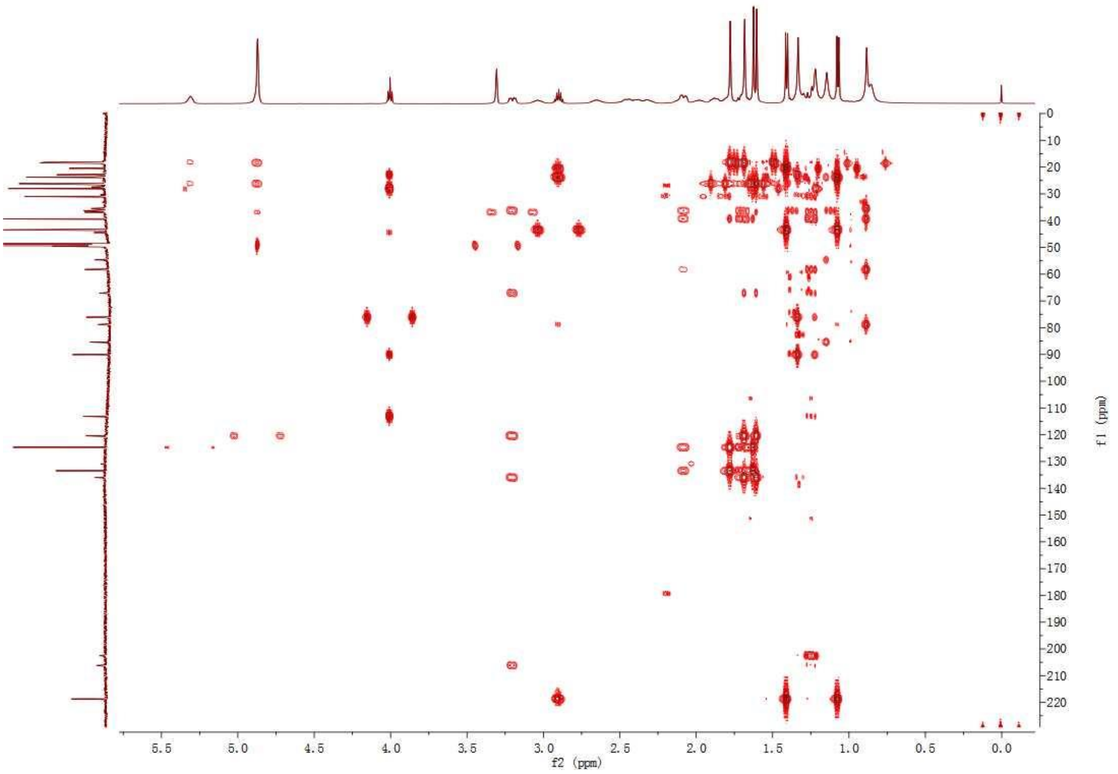


**Figure S10.** The ROESY spectrum of Hyperacmosin R (**1**).

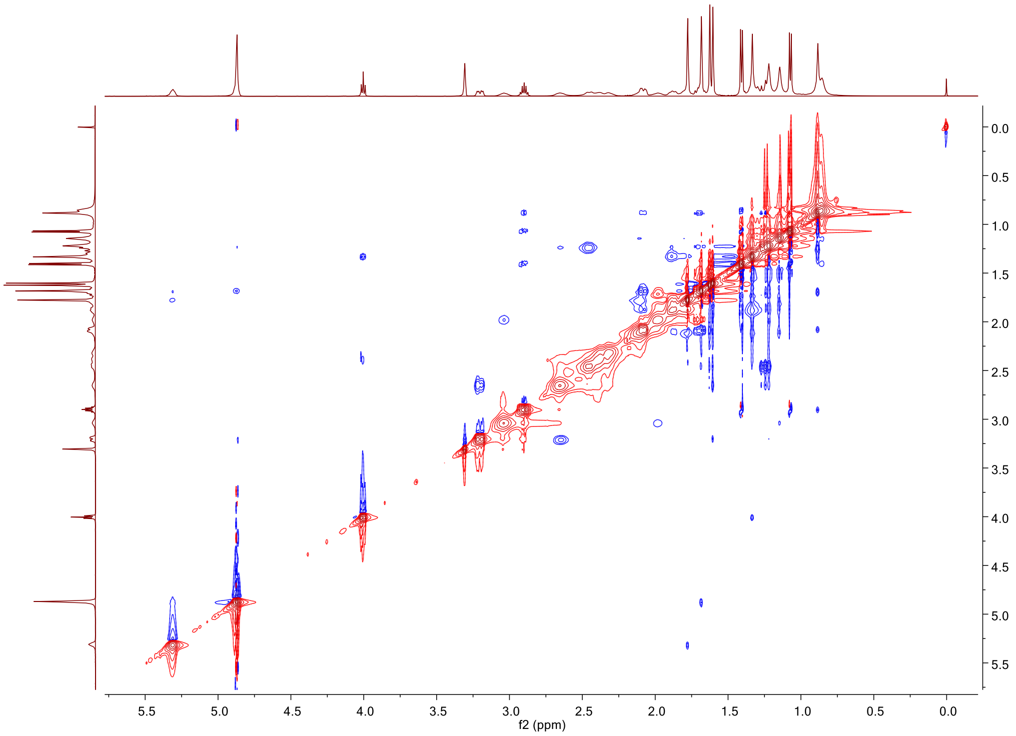

**Figure S11.** The Experimental ECD spectrum of Hyperacmosin R (**1**).

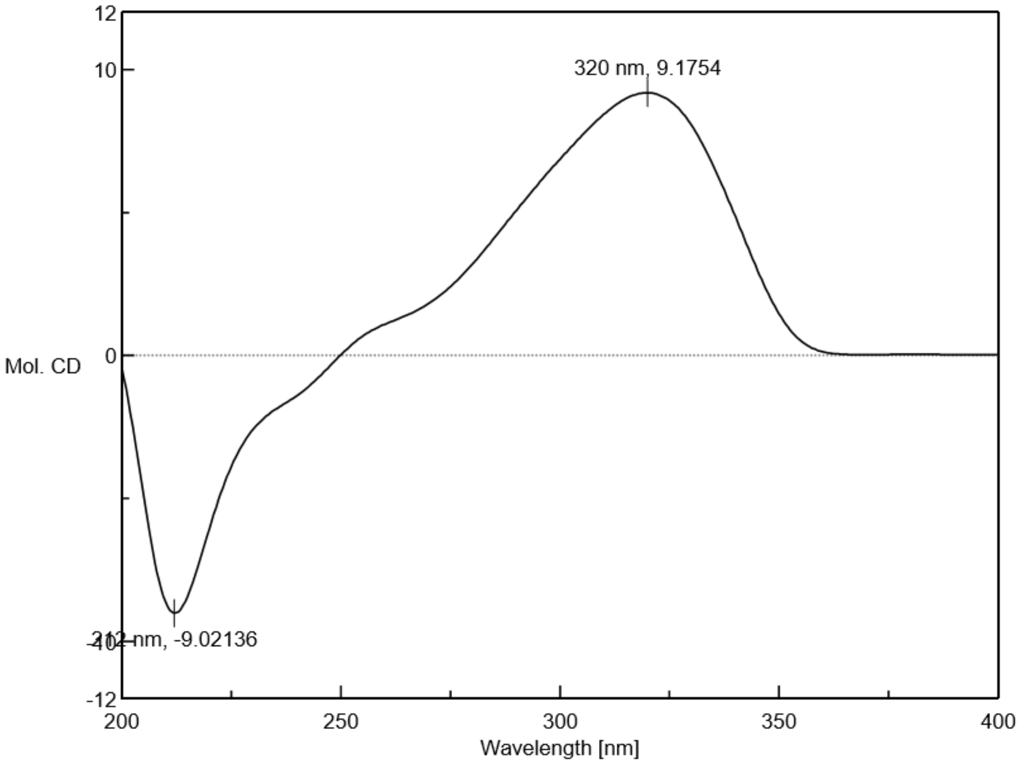


**Figure S12.** The HRESIMS spectrum of Hyperacmosin S (**2**).

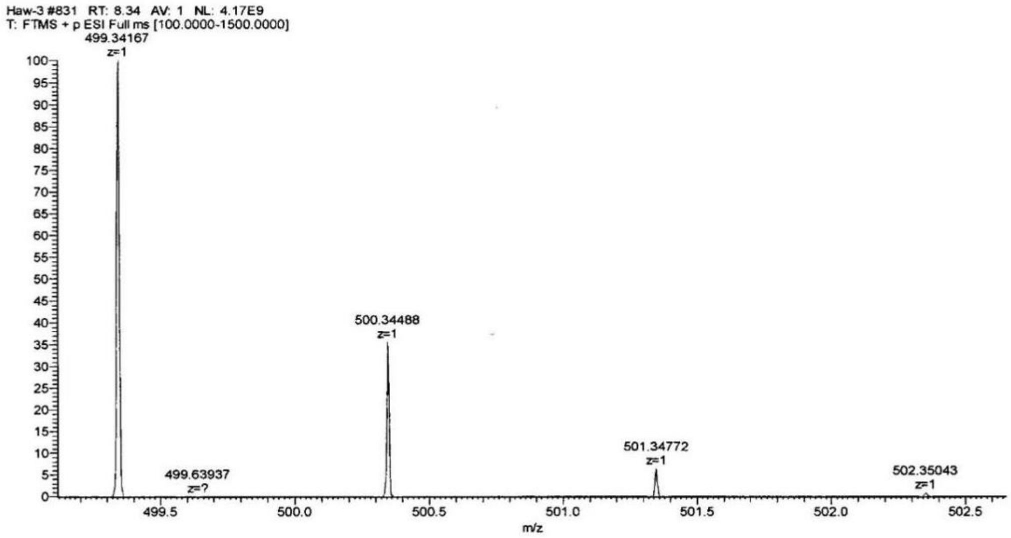


| m/z | Theo. Mass | Delta (ppm) | RDB equiv. | Composition | |
|---|---|---|---|---|---|
| | | | | | |
| 499.34167 | 499.34180 | -0.26 | 8.5 | C31 H47 O5 | M+H |

**Figure S13.** The UV spectrum of Hyperacmosin S (**2**).

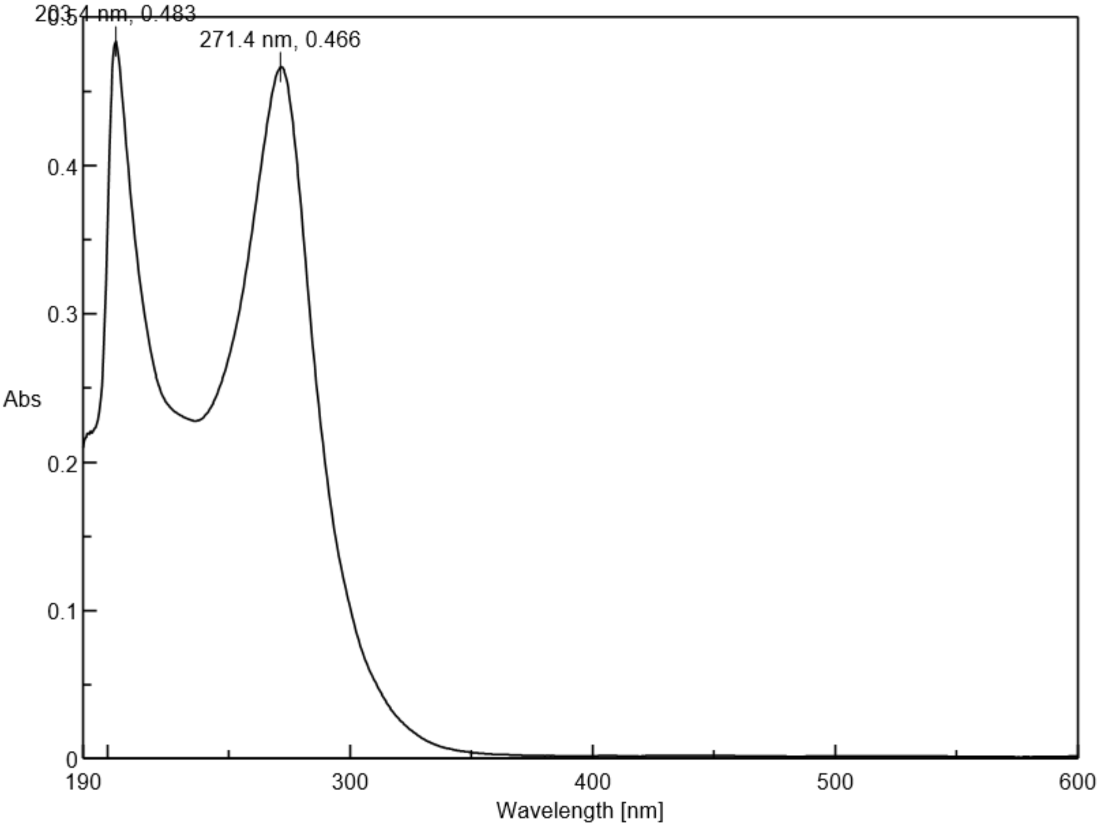


**Figure S14.** The IR spectrum of Hyperacmosin S (**2**).

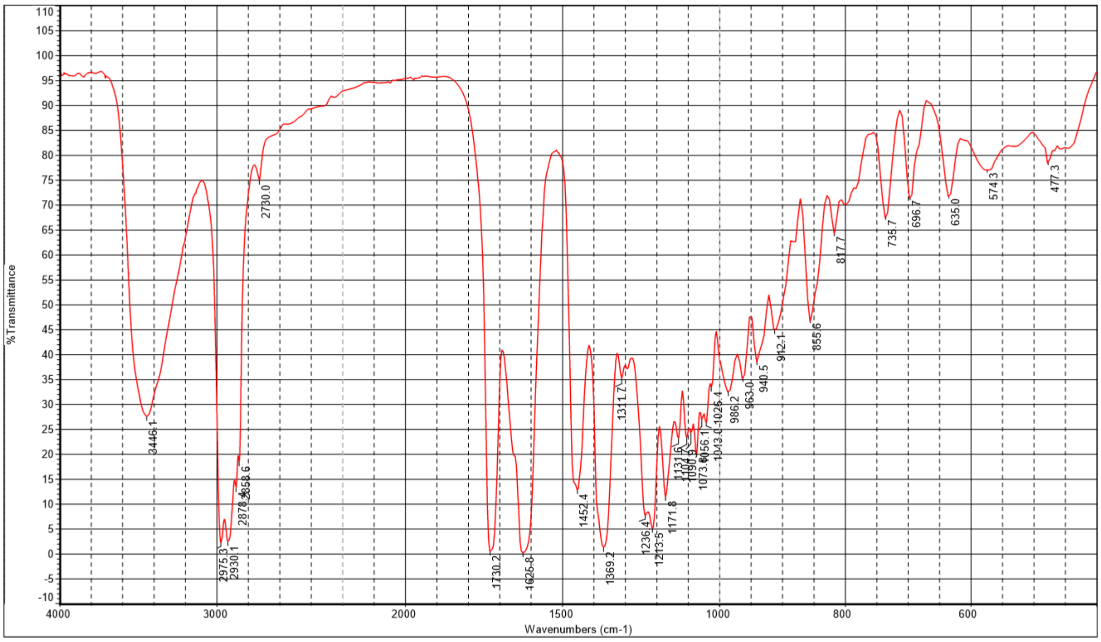

**Figure S15.** The $^{1}$H NMR spectrum of Hyperacmosin S (**2**) in $CDCl_3$ (400 MHz).

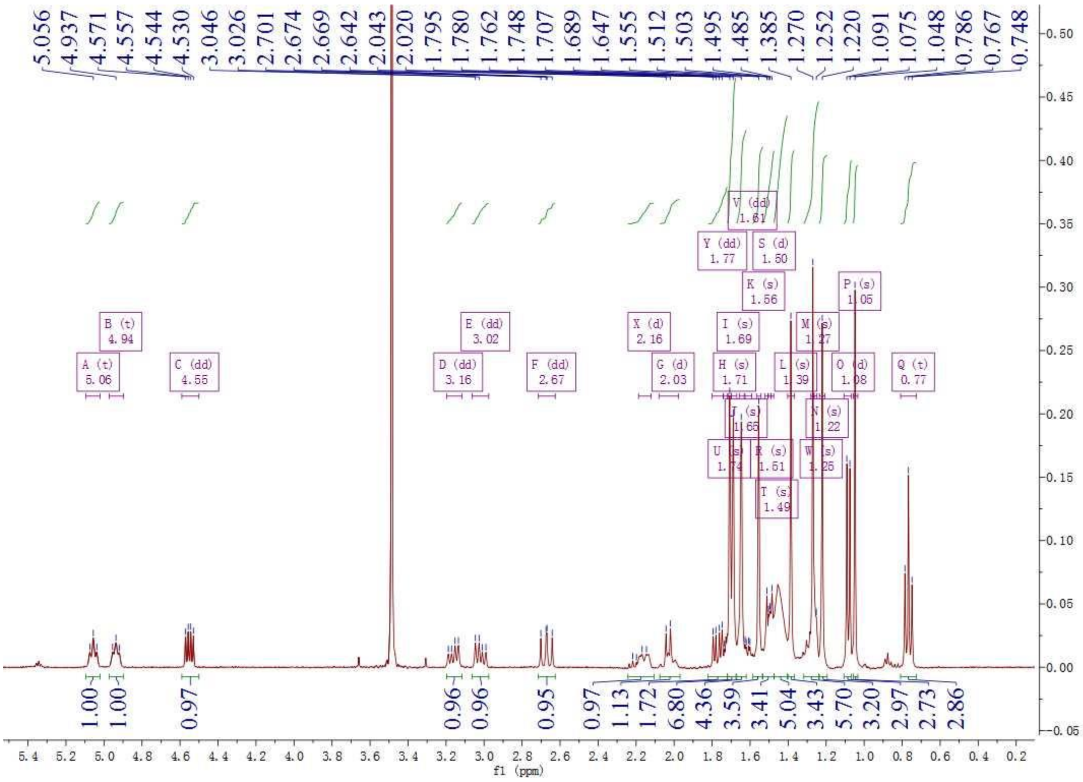

**Figure S16.** The $^{13}$C NMR spectrum of Hyperacmosin S (**2**) in $CDCl_3$ (125 MHz).

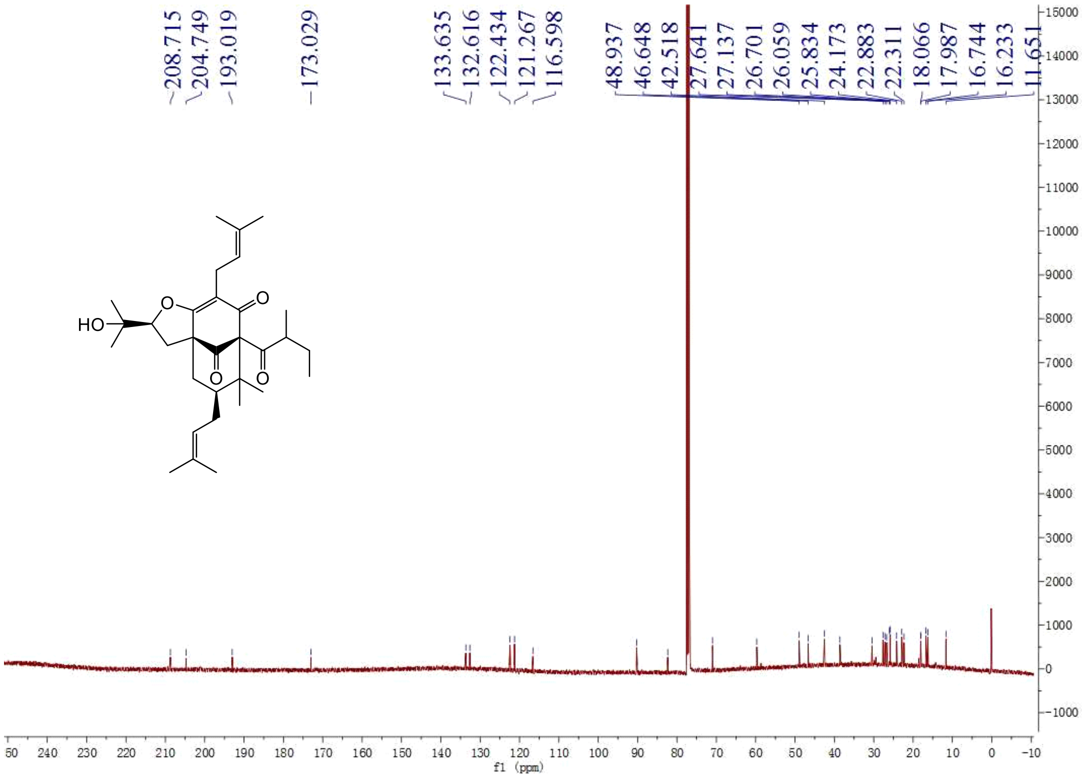

**Figure S17.** The HSQC spectrum of Hyperacmosin S (**2**).

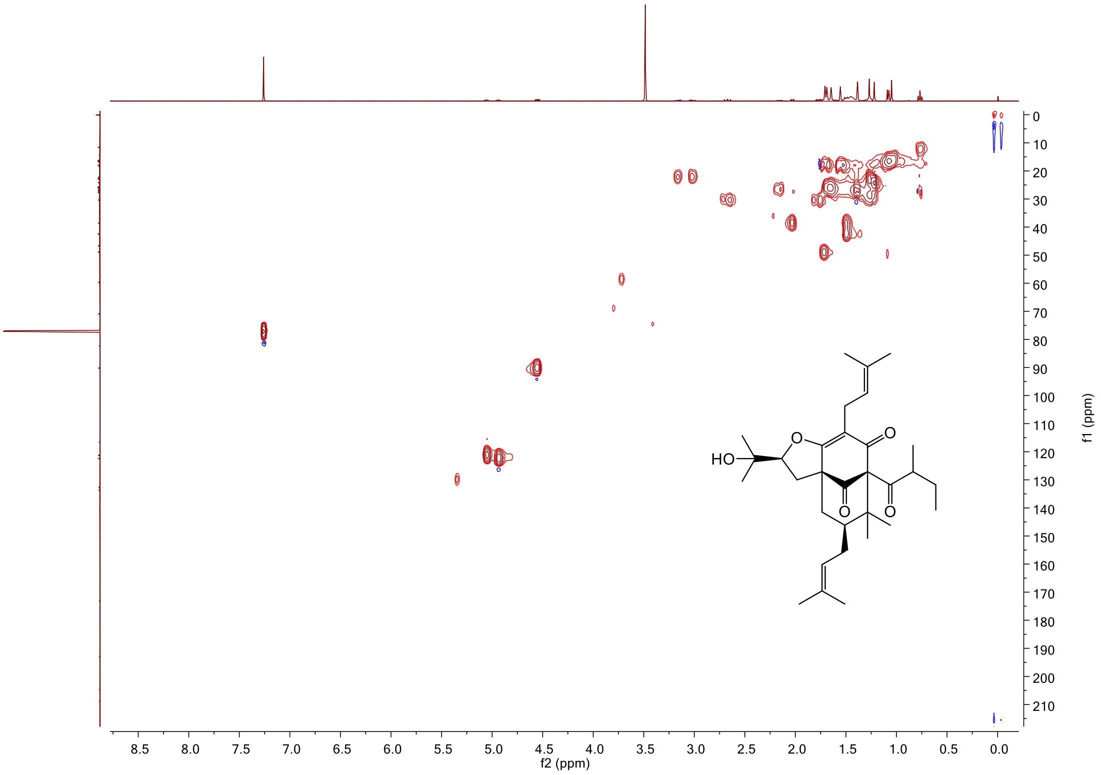


**Figure S18.** The HMBC spectrum of Hyperacmosin S (**2**).

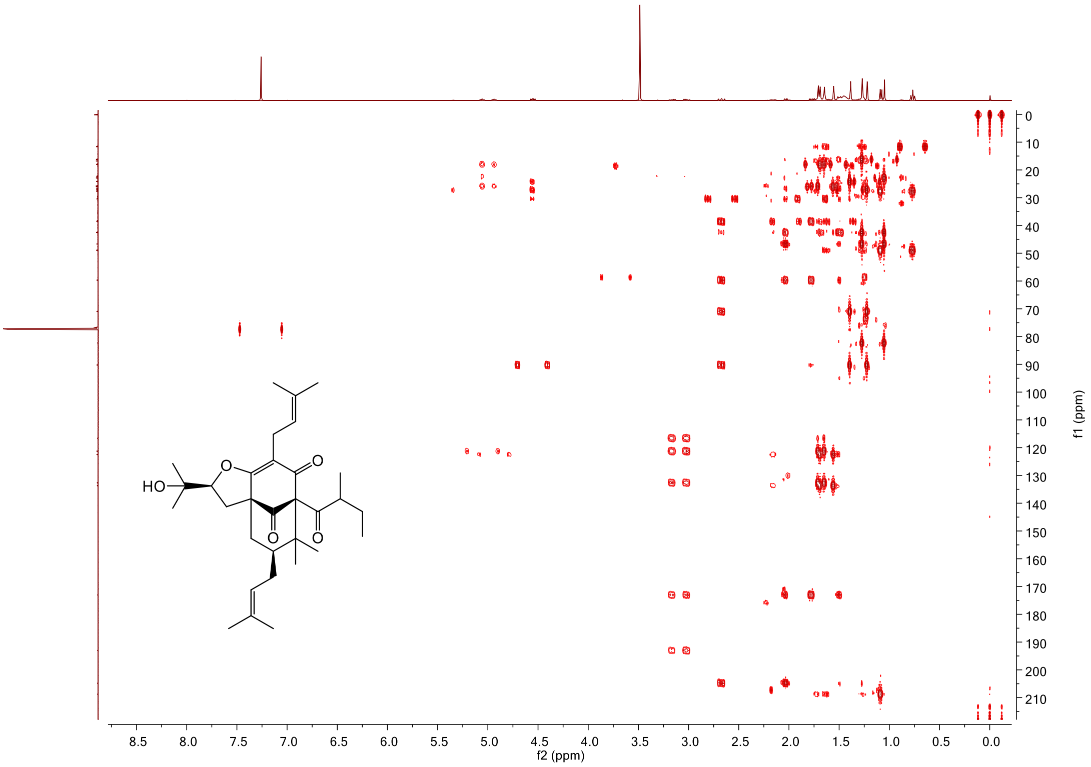

**Figure S19.** The ROESY spectrum of Hyperacmosin S (**2**).

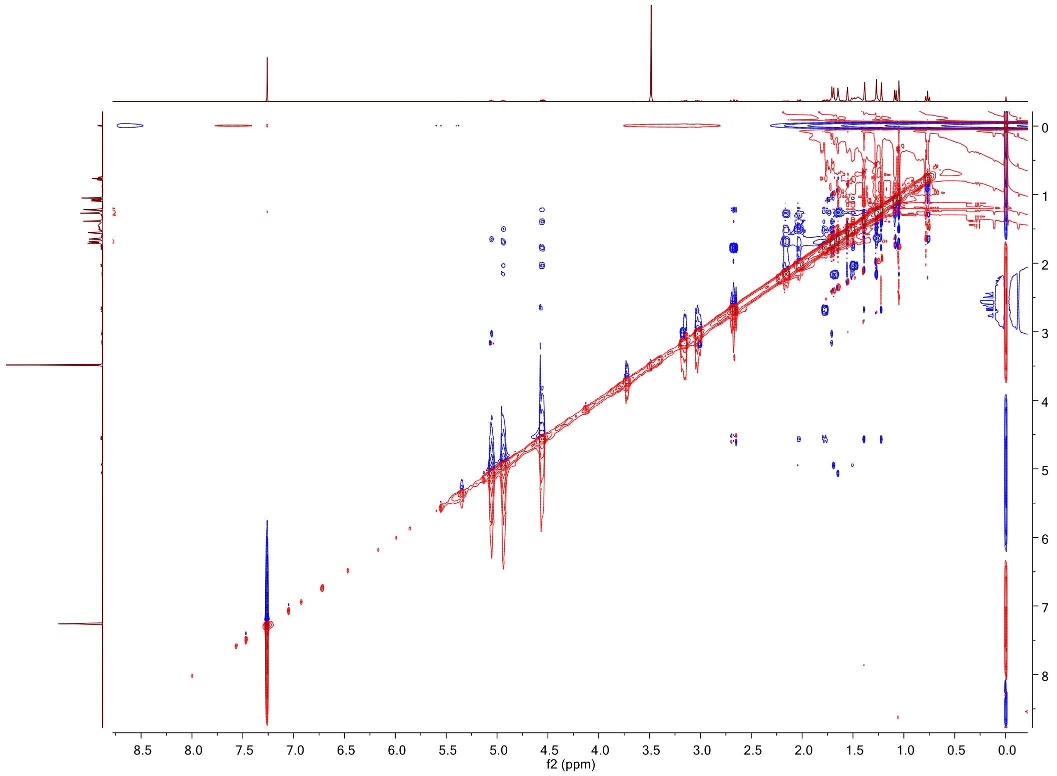


**Figure S20.** The Experimental ECD spectrum of Hyperacmosin S (**2**).

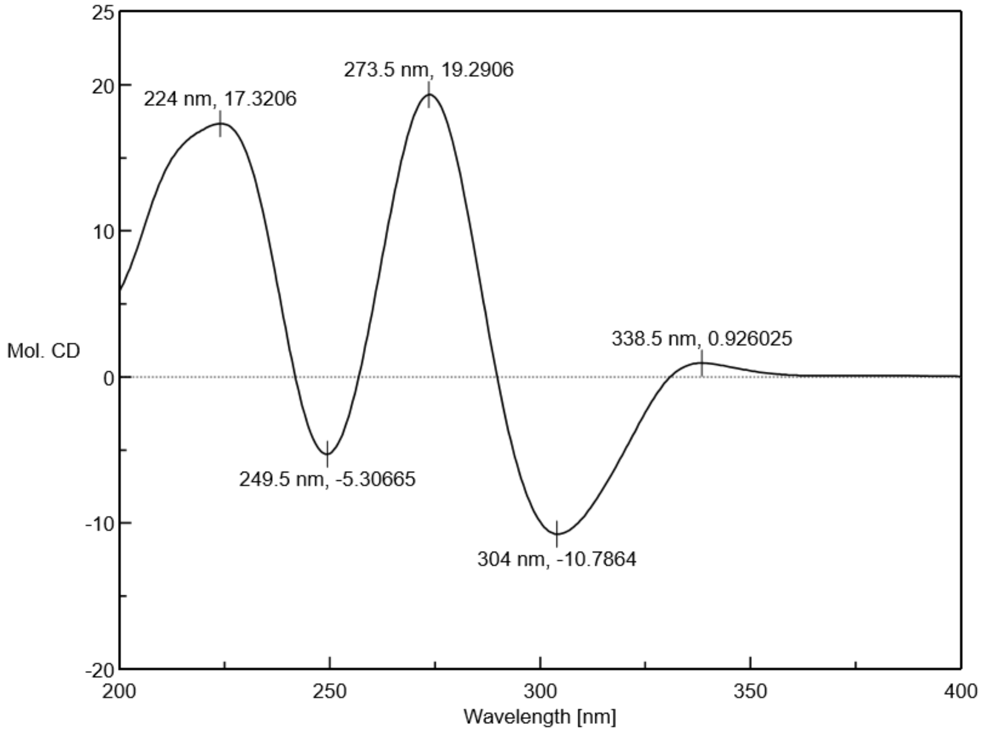

**Figure S21.** The flow chart for the separation of compounds **1-11**.

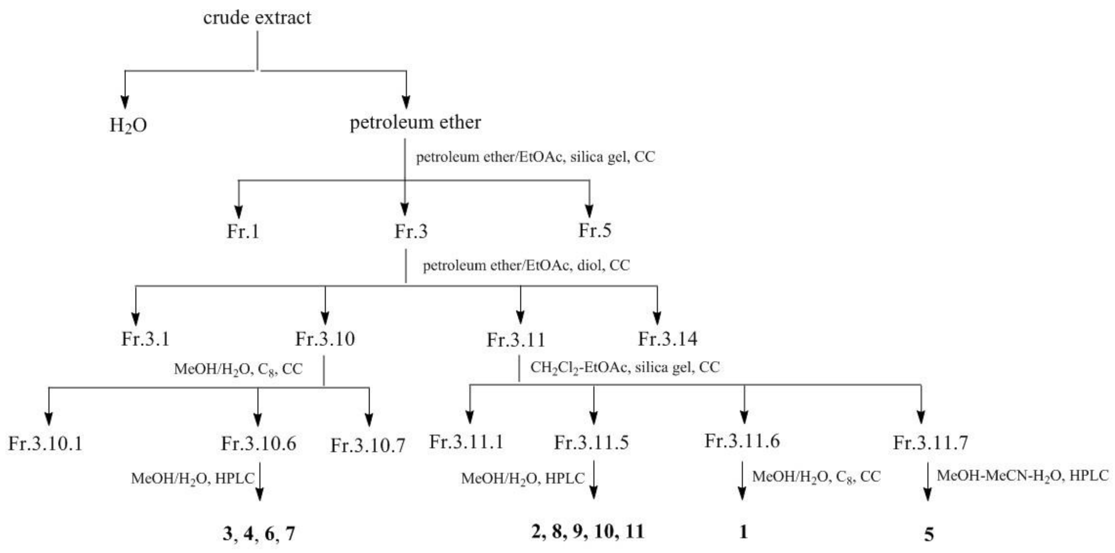

Supplement: Supplementary file 1 [file molecules-27-05932-s001.zip › molecules-1880881-supplementary.pdf]
